# Are Lifestyle Interventions in Primary Care Cost-Effective? – An Analysis Based on a Markov Model, Differences-In-Differences Approach and the Swedish Björknäs Study

**DOI:** 10.1371/journal.pone.0080672

**Published:** 2013-11-14

**Authors:** Sanjib Saha, Katarina Steen Carlsson, Ulf-G Gerdtham, Margareta K. Eriksson, Lars Hagberg, Mats Eliasson, Pia Johansson

**Affiliations:** 1 Centre for Primary Healthcare Research, Skåne University Hospital, Malmö, Lund University/Region Skåne, Malmö, Sweden; 2 Health Economics and Management, Institute of Economic Research, Lund University, Lund, Sweden; 3 Economics Department, Lund University, Lund, Sweden; 4 Department of Public Health and Department of Research, Norrbotten County Council, Luleå, Sweden; 5 Department of Health Science, Luleå University of Technology, Luleå, Sweden; 6 Centre for Healthcare Science, Örebro County Council and Örebro University, Örebro, Sweden; 7 Department of Public Health and Clinical Medicine, Sunderby Research Unit, Umeå University, Umeå, Sweden; 8 Centre for Health Economics, Informatics and Healthcare Research, Stockholm County Council, Stockholm, Sweden; University of Milan, Italy

## Abstract

**Background:**

Lifestyle interventions affect patients’ risk factors for metabolic syndrome (MeSy), a pre-stage to cardiovascular diseases, diabetes and related complications. An effective lifestyle intervention is the Swedish Björknäs intervention, a 3-year randomized controlled trial in primary care for MeSy patients. To include future disease-related cost and health consequences in a cost-effectiveness analysis, a simulation model was used to estimate the short-term (3-year) and long-term (lifelong) cost-effectiveness of the Björknäs study.

**Methodology/ Principal Findings:**

A Markov micro-simulation model was used to predict the cost and quality-adjusted life years (QALYs) for MeSy-related diseases based on ten risk factors. Model inputs were levels of individual risk factors at baseline and at the third year. The model estimated short-term and long-term costs and QALYs for the intervention and control groups. The cost-effectiveness of the intervention was assessed using differences-in-differences approach to compare the changes between the groups in the health care and societal perspectives, using a 3% discount rate. A 95% confidence interval (CI), based on bootstrapping, and sensitivity analyses describe the uncertainty in the estimates. In the short-term, costs are predicted to increase over time in both groups, but less in the intervention group, resulting in an average cost saving/reduction of US$-700 (in 2012, US$1=six point five seven SEK) and US$-500, in the societal and health care perspectives. The long-term estimate also predicts increased costs, but considerably less in the intervention group: US$-7,300 (95% CI: US$-19,700 to US$-1,000) in the societal, and US$-1,500 (95% CI: US$-5,400 to US$2,650) in the health care perspective. As intervention costs were US$211 per participant, the intervention would result in cost saving. Furthermore, in the long-term an estimated 0.46 QALYs (95% CI: 0.12 to 0.69) per participant would be gained.

**Conclusions/ Significance:**

The Swedish Björknäs study appears to reduce demands on societal and health care resources and increase health-related quality of life.

## Introduction

Lifestyle interventions with healthy food habits and increased physical exercise have been shown to be effective in the treatment and prevention of metabolic syndrome (MeSy) [1–3], which is a cluster of risk factors for cardiovascular diseases and type 2 diabetes as well as all-cause mortality [4,5]. Metabolic syndrome is a global public health problem with a prevalence of 34% in the USA [6], 23–25% in European countries [7] and close to 25% for middle-aged people in Sweden [8,9].

High-quality randomized clinical trials (RCTs) focusing on lifestyle interventions for MeSy patients are rare, especially in primary care [10]. One such intervention is the Swedish Björknäs intervention [11,12]. This 3-year group-based intervention achieved statistically significant differences on several risk factors for MeSy, for example blood pressure and waist circumference as well as self-reported time spent on physical activity [12].

Besides clinical effectiveness, a further aspect that needs to be considered to assist health care decision making with scarce public health resources is the cost-effectiveness of interventions [13–15]. The Björknäs intervention was designed to evaluate the efficacy of a lifestyle intervention on cardiovascular and metabolic risk profiles [11,12]. While participants were randomized to either an intervention group or a control group, some variation in baseline individual-level characteristics posed difficulties for the cost-effectiveness analysis. For example, the intervention group had a higher proportion of 50+-year-old participants (80% v. 66%, p<0.05), which has affected the calculation of health gains. A standard method for adjusting for baseline variation is the differences-in-differences (DD) approach [16], which is widely used in impact evaluation in economic analyses of the labour market [17,18] and also in the medical field [19].

Cost-effectiveness analyses of lifestyle interventions are more complicated than evaluations of treatment where all important health effects can be expected to manifest in the short term. This is because lifestyle interventions affect many diseases such as diabetes [20], cardiovascular diseases [21], certain types of cancer [22], body pain [23], mental health [24], etc. Furthermore, in contrast to the effect of surgery or a drug therapy, with lifestyle interventions it is uncertain whether a change in behaviour persists. Nevertheless, informed decision making demands that available data are analysed and uncertainties are described. In this respect, the focus can be on either the treatment effect, where the lifestyle intervention reduces the risk factors of MeSy, or the preventive effect, where reduction of risk factors reduces future disease events. A within-trial cost-effectiveness analysis of the Björknäs intervention using a (3-year) before-after design has been published [25] concentrating on the treatment effect; however, cost-effectiveness studies focusing on preventive effects and long- term results remain to be conducted.

In general, within-trial cost-effectiveness analysis has several limitations [26], which include a limited time horizon, small sample size, failure to incorporate all the evidence, lack of relevance to the decision context and limited opportunity to quantify decision uncertainty. For lifestyle interventions, limited time horizon underestimate the benefits, as studies have shown that intervention effects persist after the trial period [27,28]. Therefore, within-trial cost-effectiveness analysis may provide biased estimation. Moreover, uncertainty around the duration of the effect of the lifestyle intervention, which is a driving force of intervention cost-effectiveness, according to a recent review [10], is impossible to accommodate in the within-trial perspective. 

An alternative to within-trial analyses are analyses based on simulation models which capture also the effects of the intervention beyond the trial period. Moreover, these enable reviewing uncertainty around the effectiveness of the intervention. However, cost-effectiveness analyses of lifestyle interventions by simulation models show inconclusive results [29–33]. 

In this study we used a simulation model to evaluate the cost-effectiveness of the Swedish Björknäs intervention using DD approach. We estimated the cost-effectiveness for two different time periods: the short term, to compare within-trial cost-effectiveness performed previously (but without having taken account of differences in baseline characteristics), and the long term, to capture all the expected consequences in costs and health effects of this lifestyle intervention from a health care and societal perspective. 

## Materials and Methods

### The intervention

Details about the Swedish Björknäs intervention have been published elsewhere [11,12]. In brief, the Björknäs study was a 3-year RCT with patients recruited in primary care centres of Björknäs. Using computer-generated random numbers the researchers randomly allocated participants to an intervention and a control group. The intervention consisted of physiotherapist-supervised physical exercise three times a week and diet counselling on five occasions for the first 3 months, followed by regular group meetings. The diet counselling followed the Nordic Nutrition Recommendations and consisted of both verbal and written guidelines [11]. After the 3 months of active intervention, participants were invited to attend group meetings six times in the first year, four times in the second year and twice in the third year. The control group received verbal and written information about physical exercise and dietary recommendations in one single meeting.

### Participant information

The characteristics of the participants, i.e. anthropometric, physiological and self-reported physical activity, were recorded at baseline and in the first, second and third year. Table 1 shows characteristics of 145 persons (intervention n=71; control n=74) agreeing to participate at baseline and later measurements. The overall drop-out rate during the intervention period was 17% and 58 and 62 participants were available at the third year follow-up in the intervention and control groups, respectively [12].

**Table 1:** Participants’ characteristics at baseline, and at 1st year and 3rd year.

**Variables**	**Baseline**		**1st year**		**3rd year**	
	**Control (n=74)**	**Interven-tion (n=71)**	**Control (n=63)**	**Interven-tion (n=60)**	**Control (n=62)**	**Interven-tion (n=58)**
Age, yrs^a^	53.1 (8.2)	55.7 (6.6)	55.1 (6.8)	57.0 (5.8)	57.2 (6.8)	59.2 (5.8)
Male^b^	27 (36.5)	35 (49.3)	20 (31.7)	32 (53.3)	20 (32.3)	31 (53.4)
Female^b^	47 (63.5)	36 (50.7)	43 (68.3)	28 (46.7)	42 (67.7)	27 (46.6)
Systolic blood pressure (mmHg)^a^	144.7 (17.6)	145.6 (15.5)	143.8 (15.5)	141.0 (13.2)	147.3 (14.8)	141.6 (14.0)
BMI^a^	29.4 (5.1)	30.1 (5.2)	28.3 (4.7)	29.2 (4.7)	28.5 (5)	29.5 (4.8)
HbA1c (%)^a^	6.62 (2.05)	6.30 (1.35)	6.3 (1.1)	5.9 (1.7)	6.9 (1.5)	6.4 (1.9)
Fasting blood glucose (mmol/L)^a^	5.20 (0.05)	5.24 (0.05)	5.3 (0.6)	5.2 (0.9)	5.5 (0.8)	5.4 (0.9)
High-density lipoprotein (mmol/L)^a^	1.46 (0.4)	1.39 (0.3)	1.5 (0.4)	1.3 (0.3)	1.4 (0.4)	1.3 (0.3)
Total cholesterol (mmol/L)^a^	5.4 (0.9)	5.4 (1.0)	5.5 (0.8)	5.7 (1.1)	5.3 (1)	5.4 (1)
Diabetes^b^	17 (23)	23 (32)	13 (20.6)	20 (33.3)	13 (21)	20 (34.5)
Duration of diabetes (yrs)^c^	5 (4.3)	7 (5.8)	5 (3.5)	6.5 (5.6)	8 (3.5)	9.5 (5.6)
Smokers^b^	13 (17.6)	17 (24)	9 (14.3)	11 (18.3)	7 (11.3)	6 (10.3)

^a^ mean (SD), ^b^ number of observations (%), ^c^ median (SD)

BMI = body mass index; HbA1c = glycated haemoglobin; SD = standard deviation

### Simulation model

**Figure 1:**
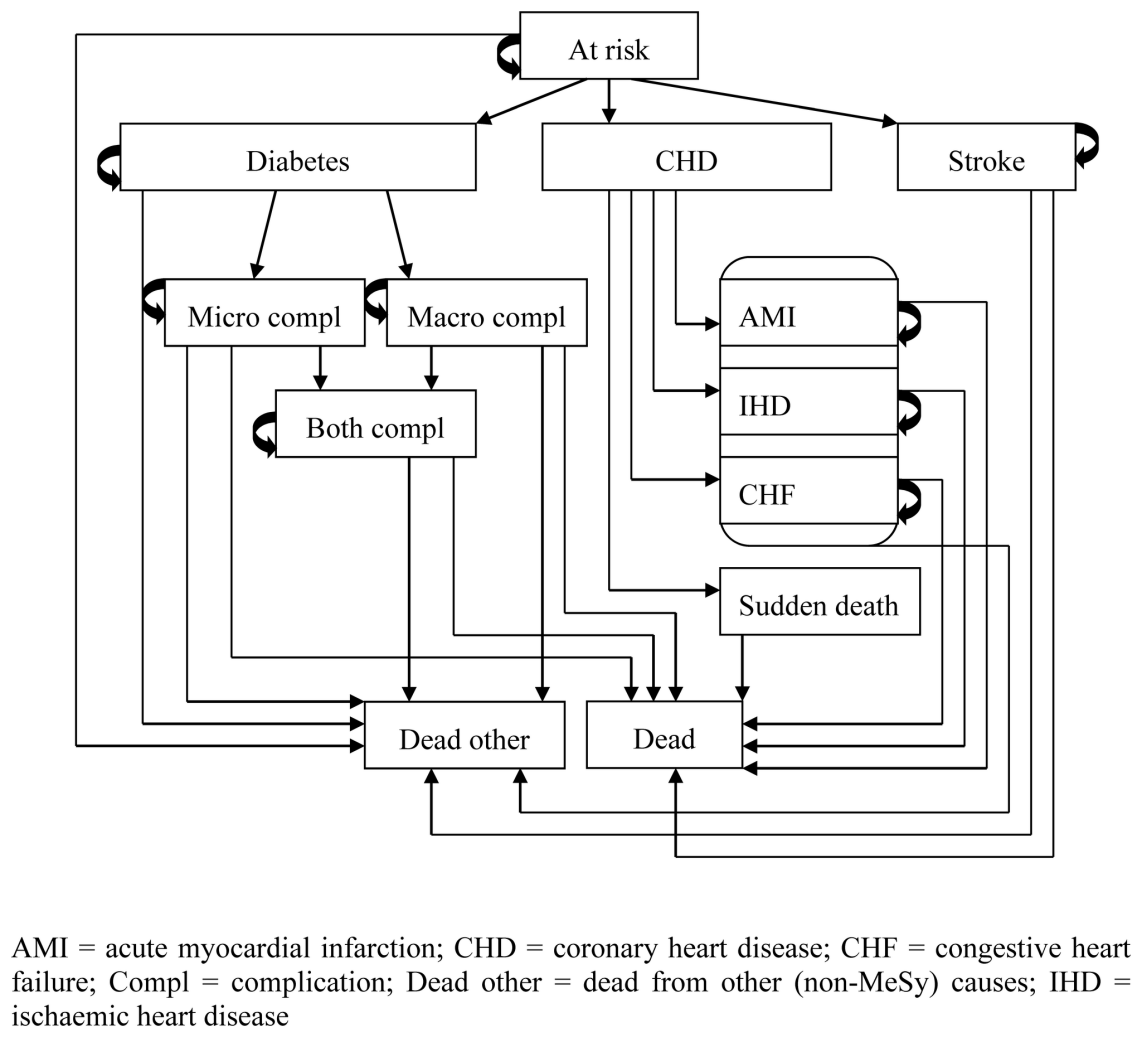
State-transition diagram.

We used an updated version of a Markov micro-simulation model for MeSy, previously used for cost-effectiveness analyses [34,35]. The model incorporated the core diseases due to MeSy: cardiovascular diseases, type 2 diabetes and related complications (Figure 1). The simulations were based on the levels of MeSy risk factors, body mass index (BMI), smoking habits, systolic blood pressure, high-density lipoprotein (HDL), fasting plasma glucose, glycated haemoglobin (HbA1c), and presence and duration of diabetes, together with gender and age. The model risk equations for developing cardiovascular diseases were from The Framingham Heart Study [36], diabetes from the San Antonio Heart Study [37], and complications of diabetes from the UKPDS study [38]. Further information can be found in the model technical report, including sources and a discussion of the model uncertainty and validity [39] (see supporting information, File S1). The costs of diseases and utility weights were taken from published Swedish studies, where available. The costs included medical treatment costs, costs for institutional health care, pharmaceuticals, informal care and other costs for patients and relatives, and productivity loss due to morbidity. Transitions between health states could occur once a year. The termination age was set at 85 years. The model was developed using Treeage Pro 2009 software (Treeage Software Inc, Williamstown, MA, USA).

The model was run for each of the participants in both groups (the control group and the intervention group), with participants’ individual characteristics as micro-simulation, and with 10,000 repetitions done. At the beginning of the simulations, participants were considered to be in the health state “at risk” (Figure 1), with the exception of participants diagnosed with diabetes. They started in the health state “diabetes”, and for them, the number of years with diabetes was included as an individual characteristic. Thereafter, based on each participant’s individual characteristics and risk factor levels, the participants were allowed to move to later health states according to disease progression (Figure 1). Each time period they spent in a health state, say myocardial infraction, the model accrued a cost and QALY, which were summed at the end of the simulation. The model thus resulted in estimates of the expected costs and the average number of QALYs for each participant, based on 10,000 simulations. For the long-term analysis, we ran the model for each participant from their baseline age and for life (i.e. until the termination age), while for the short-term analysis, the model covered 3 years, i.e. the trial period duration.

### Perspectives of analysis

We performed analyses both from the health care and from the societal perspective. The societal perspective implies that all costs are included irrespective of who is burdened by them, while the health care perspective is concerned only with resource use within by the health care sector [40]. In this analysis we included medical treatment costs, pharmaceutical costs and costs for community care for each disease in the model (Supporting information, File S1).

### Differences-in-differences approach

The DD approach [16] accounts for baseline differences between two groups and measures the differences in trends between two groups over time. The change over time was calculated for each group by comparing observed outcomes in “with study” with a counterfactual “reference” scenario, leading to the within-group differences. The model was first run with each participant’s characteristics from the “with study” scenario, and then with the “reference” scenario. For example, the simulation of “with study” assumed third-year observed risk factor levels as start values while keeping age at baseline values. The counterfactual “reference” scenario without the effect of intervention used baseline risk factor levels. Thereafter, the group-based average cost and QALYs were calculated for each scenario. The differences were calculated, the first difference concerning within-group comparisons over time and the second difference concerning between-group comparisons over time.

### Cost-effectiveness analysis

We report the incremental cost-effectiveness ratio (ICER) which measures the incremental cost divided by the incremental benefit. The incremental cost is the difference in the expected impact on costs, using DD approach, between the intervention and the control group including intervention costs. The incremental benefit is the difference in the expected impact on quality of life using DD approach. The programme cost for the Björknäs intervention was US$211 for each participant (retrieved from the within-trial cost-effectiveness analysis of the Björknäs intervention [25]). All costs were converted to 2012 prices, according to the Swedish consumer price index [41], and then further converted to US$. The base case analysis used a 3% discount rate for costs and QALYs. Sweden does not have a formal threshold for cost-effectiveness ratios although the Swedish National Board of Health and Welfare have considered interventions costing <five hundred thousand SEK per QALY gained (76,000 US$/QALY) as cost-effective [42]. The Pharmaceutical Benefits Board considers disease severity and availability of alternative treatments for granting subsidies for drugs and medical devices. The World Health Organization regards an ICER lower than gross domestic product per capita of US$57,114 [43] as very cost-effective [44].

We used participants’ first-year and third-year characteristics for the model simulation. The base case assumptions for modelling were that the third-year characteristics of participants would remain constant until they died or reached the termination age in the long-term analysis, and for 3 years in the short-term analysis.

In the base case analysis, the outcomes were estimated on an intention-to-treat basis; including all 145 participants who started the Björknäs intervention. For missing follow-up data, we carried forward the last observation. To reflect the uncertainty in the estimates, we performed non-parametric bootstrap analysis with 1,000 samples using SPSS, version 20 (SPSS Inc., Chicago, IL, USA), to calculate the 95% CI for the estimators used in the DD.

### Sensitivity analysis

We performed several univariate analyses and one multivariate sensitivity analysis to reflect the uncertainty in the results. The analyses were performed for the long-term analyses.

#### Intervention effect assumption

In the base case analysis, we assumed that the third-year risk factor levels would remain until death, i.e. the effect of the intervention would last for the full remaining life span of the participants. This is an optimistic assumption [10] and consequently we also performed sensitivity analyses assuming that the risk factor levels would return to baseline at 10 years (analysis 1a) and 5 years (analysis 1b) after the intervention terminated. We also assumed that the behaviour of the participants would immediately return to baseline levels (analysis 1c) after the intervention terminated, which was a pessimistic assumption. We further studied the assumption that the first-year risk factor levels would remain until death (analysis 1d).

#### Missing cases and complete cases

Missing data are generally dealt with in clinical studies by carrying forward the last observation, as was done in the base case of this Björknäs intervention [45]. As an alternative method, we imputed all the missing data of the participants’ characteristics both in the control group and in the intervention group using multiple imputations (MIs) [46]. The major advantage of MI was that it produced standard errors that reflected both within- and between-sample variations. We generated ten different datasets for each group in SPSS, version 20, for both the first year and the third year using Markov chain Monte Carlo method. All available data on age, sex, blood pressure, BMI, total cholesterol, HDL, fasting glucose level, HbA1c, presence of diabetes, duration of diabetes and smoking status were employed to impute the missing data points. Thereafter, the multiple datasets were analysed and pooled estimates were computed following the method described by Rubin [47] (analysis 2a).. We also performed a subgroup analysis of participants who had completed the intervention, 62 in the control group and 58 in the intervention group(analysis 2b).

#### Subgroup analysis for diabetes

Results are reported separately for persons with and without diabetes at baseline (analyses 3a,b).

#### Time horizon

We increased the model termination age from 85 years to 120 years (analysis 4).

####  Swedish data sources

For the multivariate sensitivity analysis, we used data from previously published Swedish studies. For example, risks for diabetes were based on the Stockholm Diabetes Prevention Program (personal communication), risks for stroke and myocardial infraction on the NORA study [48], etc. Details of the data sources were reported in the technical report (supporting information, File S1) (analysis 5).

#### Study group mean value

In this analysis, we used the mean characteristics of the participants and did not consider participants’ heterogeneity (analysis 6).

#### Discount rate

The analysis included no discounting and a 5% discount rate on costs and QALYs (analyses 7a,b).

## Results

### Base case analysis

Table 2 presents the main results for the short-term and long-term. In the short-term analysis, both the intervention group and the control group were estimated to have increased the cost but the increase in cost was higher for the control group compared with the intervention group. The DD for cost was US$-700 (95% CI: US$-1,100–US$-260) from the societal perspective and US$-500 (95% CI: US$-1,000–US$-200) from the health care perspective. The model-based analysis using DD found no difference in QALYs. Therefore, in the short term, the Björknäs intervention only saved cost but did not lead to any gains in QALYs. 

**Table 2:** Base case results regarding mean costs (in US$, 2012 price year) and quality-adjusted life years (QALYs) per participant in the short and long term. ^*^.

Model simulation	Perspectives	Predicted cost (US$) (95% CI by bootstrapping)		Predicted QALY (95% CI by bootstrapping)	
		Intervention	Control	Intervention	Control
Short-term, baseline –year 3	**Societal**				
	With Study	14,000 (10,400 to 17,600)	11,050 (8,200 to 14,100)	2.27 (2.247 to 2.298)	2.31 (2.285 to 2.33)
	Reference	13,950 (10,500 to 17,500)	10,300 (7,400 to 13,300)	2.27 (2.250 to 2.299)	2.31 (2.287 to 2.335)
	Difference	50 (-300 to 400)	750 (450 to 1,100)	0.00 (-0.008 to -0.000)	0.00 (-0.004 to 0.00)
	**DD**	**-700 (-1,100 to -260)**		**0.00 (-0.007 to 0.001)**	
	**Health care**				
	With Study	9,530 (7,000 to 12,100)	7,260 (5,300 to 9,450)	2.27 (2.247 to 2.298)	2.31 (2.285 to 2.335)
	Reference	9470 (7,000 to 12,000)	6,700 (4,600 to 8500)	2.27 (2.250 to 2.299)	2.31 (2.287 to 2.335)
	Difference	60 (-190 to 300)	560 (400 to 950)	0.00 (-0.008 to -0.000)	0.00 (-0.004 to 0.003)
	**DD**	**-500 (-1,000 to -200)**		**0.00 (-0.007 to 0.001)**	
Long-term, baseline - death	**Societal**				
	With Study	75,500 (67,000 to 83,570)	82,800 (47,900 to 94,300)	9.83 (9.108 to 10.671)	10.81 (10.044 to 11.582)
	Reference	75,200 (67,900 to 82,400)	75,200 (42,850 to 85,100)	9.80 (9.054 to 10.553)	11.24 (10.544 to 11.907)
	Difference	300 (-3,660 to 4,600)	7,600 (6,200 to 16,900)	0.03 (-0.243 to 0.311)	-0.43 (-0.684 to -0.235)
	**DD**	**-7,300 (19,700 to -1,000**)		**0.46 (0.116 to 0.69)**	
	**Health care**				
	With study	58,400 (53,200 to 63,700)	59,000 (53,000 to 66,200)	9.83 (9.108 to 10.671)	10.81 (10.044 to 11.582)
	Reference	58,000 (52,600 to 63,600)	57,100 (51,000 to 65,000)	9.80 (9.054 to 10.553)	11.24 (10.544 to 11.907)
	Difference	400 (-1,700 to 2,400)	1,900 (-1,900 to 5,000)	0.03 (-0.243 to 0.311)	-0.43 (-0.684 to -0.235)
	**DD**	**-1,500 (-5,400 to 2,650)**		**0.46 (0.116 to 0.69)**	

* Cost figures are rounded

In the long-term analysis, the result was similar. There was an increase in cost in the “with study” scenario compared with the “reference” scenario where the cost increase was higher in the control group compared with the intervention group. The DD was US$-7,300 (95% CI: US$-19,700–US$-1,000) from the societal perspective and US$-1,500 (95% CI: US$-5,400–US$2,650) from the health care perspective. Regarding QALYs in the long term, the control group is estimated to have their QALYs reduced by -0.43 (95% CI: -0.68 to -0.23) while the intervention group gained 0.03 QALYs (95% CI: -0.24 to 0.31). Therefore, the intervention group participants were estimated to gain 0.46 (95% CI: 0.11–0.69) QALYs in comparison with the control group participants. Therefore, in the long term, the intervention was estimated to reduce future societal cost and at the same time increase QALYs. Considering the programme cost of the Björknäs intervention (US$211 [25]) the intervention was cost-saving in the short and also in the long term, both from a societal perspective and from a health care perspective.

### Sensitivity analysis

The Swedish Björknäs intervention was cost-saving in the base case (Table 3). However, the results were sensitive to the assumptions of a diminishing effect of the intervention, i.e. considering whether the risk factors return to baseline (with varying time duration). If the risk factors remained for 10 years (analysis 1a), the intervention was cost-saving but the cost reduction and QALY gain was lower than in the base case analysis. In the health care perspective, it was cost-effective, with US$1,152/QALY gained. The intervention was still cost-saving if the risk factors remained for 5 years after the intervention terminated, with less cost reduction and less QALY gain (analysis 1b) in the societal perspective. From the health care perspective, it was still cost-effective. If the risk factors immediately reverted to baseline values after the intervention terminated, the intervention was no longer cost-saving in either perspective (analysis 1c). There was an increase in cost but still a gain in QALYs. From the societal perspective, it required US$21,786/QALY gained and from a health care perspective it required US$7,613/QALY including programme costs, which is considered cost-effective in Sweden. Assuming the first-year risk factors would remain the same, the intervention was cost-saving from a societal perspective but not from a health care perspective. From a health care perspective, there was an increase in cost of US$90 with 0.128 QALY gains, which was cost-effective (analysis 1d).

**Table 3:** Sensitivity analysis, and difference in cost (in US$, 2012 price year) and quality-adjusted life years (QALYs) in the long term, with different perspectives shown.

No.	Situation	Cost (US$)		QALY	Interpretation	
		Societal perspective	Health care perspective		Societal perspective	Health care perspective
	**Base case** (3rd-year risk factors remain until death)	-7,300	-1,500	0.46	Cost-saving	Cost-saving
1a.	Risk factors return to baseline after 10 years	-930	250	0.40	Cost-saving	Cost-effective* (US$1,152/QALY )
1b.	Risk factors return to baseline after 5 years	-850	880	0.31	Cost-saving	Cost-effective* (US$3,519/QALY)
1c.	Risk factors return to baseline immediately after the intervention finishes	4,800	1,540	0.23	Cost-effective * (21,786 US$/QALY)	Cost-effective* (US$7,613/QALY)
1d.	1st-year risk factors remain until death	-1,230	90	0.12	Cost-saving	Cost-effective* (US$836/QALY)
2a.	With MIs for missing cases	-11,300	-6,100	0.47	Cost-saving	Cost-saving
2b.	Complete cases only	-10,300	-1,400	0.33	Cost-saving	Cost-saving
3a.	Only persons with diabetes	9,800	1,580	0.95	Cost-effective * (US$10,537/QALY)	Cost-effective* (US$1,885/QALY)
3b.	Only persons without diabetes	-12,900	-2,260	0.30	Cost-saving	Cost-saving
4.	Extended time horizon (max age 120 years)	-11,300	-2,370	0.27	Cost-saving	Cost-saving
5.	Swedish data sources	-780	-160	0.04	Cost-saving	Cost-effective* (US$1,275/QALY)
6.	Study group, mean value	-940	-1,700	0.39	Cost-saving	Cost-saving
7a.	No discounting	-14,900	-3,100	0.48	Cost-saving	Cost-saving
7b.	5% discount rate	-8,270	-1,900	0.06	Cost-saving	Cost-saving

* Including programme costs per participant (US$211); MIs = multiple imputations

The intervention was not cost-saving for the subgroup analysis of diabetic participants (analysis 3a). For diabetic participants, the QALY gain was 0.95 but there was an increase in cost, US$9,800 and US$1,580, from the societal and the health care perspective, respectively. Therefore, the intervention was cost-effective, not cost-saving, for participants with diabetes, namely US$10,537/QALY gained and US$1,885/QALY from a societal and health care perspective, respectively, including programme costs. However, for non-diabetic participants, the cost reduction was higher than in the base case analysis. The Björknäs intervention directed at diabetic participants would be cost-effective while for non-diabetic participants, it would be cost-saving. The intervention was cost-effective considering Swedish data sources from a health care perspective (analysis 5). For other sensitivity analyses, the base case result was not sensitive.

## Discussion

We estimated the cost-effectiveness of a lifestyle intervention as in the Swedish Björknäs study by model simulation for different time scales using DD approach and extensive sensitivity analyses. The analysis showed that the benefits of the intervention were reinforced in the long term when not only were costs expected to be saved but also, QALYs were expected to be gained. The cost-effectiveness result was robust to a number of changes in model assumptions, as shown by the sensitivity analysis.

The within-trial cost-effectiveness analysis of the Björknäs intervention with patient and register data found the intervention to be cost-saving [25]. Using model simulation, the intervention was cost-saving in the long term, with increased benefits compared with within-trial results. The positive impact of increasing the time horizon was in line with earlier results of the lifestyle intervention of the Diabetes Prevention Program (DPP) showing higher cost savings in the long term (US$51,600/QALY gained in the within-trial analysis [49] and US$8,800/QALY gained in the long term [30]). There were several reasons for the differences in results when comparing the DPP and the Björknäs intervention. Besides using different simulation models, the programme costs were much higher in the DPP trial, as the programme was individualized while the Björknäs intervention was group-based and conducted in a limited resource setting. The cost-effectiveness of The Finnish Diabetes Prevention Study (DPS) in Swedish settings also showed cost saving from lifetime perspectives [50]. This Björknäs intervention also evaluates a group-based intervention, but compared with the DPS we considered additional health states (coronary heart disease and congestive heart failure, and allowed diabetes to develop into micro- and macro-vascular complications) in the Markov model.

The difference between within-trial cost-effectiveness and model-based, short-term cost-effectiveness is that the within-trial calculations considered participant-reported QALYs and registered-based costs while the model-based calculations considered cost and QALYs related to disease events. One might argue that, since the within-trial cost-effectiveness of the Björknäs intervention resulted in cost-saving [25], there is no need for estimates of long-term benefits. We argue that the standard recommendation for performing cost-effectiveness analysis is to consider a longer time frame [40]. Moreover, the 3-year intervention period may be considered as a treatment for MeSy, as the participants met health professionals, interacted with them and felt motivated and managed themselves towards healthy lifestyle habits. Here the main purpose was to treat the risk factors. After the active intervention, the preventive effect of the intervention may be considered an additional benefit [51], where reduced risk factors lessen future diseases. The preventive effect may be useful for policy-making perspectives as it includes less chronic disease events in the future. Within-trial cost-effectiveness calculations showed an increase in utility weights of 0.08 (p=0.24) using the EQ-5D [25], which can be considered a treatment effect while model simulation QALY gain (0.46) can be considered a preventive effect of the Björknäs intervention. Therefore, different methods have their strength and drawbacks and it is worth mentioning that different cost-effectiveness analyses are not competitive nor is one better than the other. They complement each other and provide a broad picture of the benefits of the lifestyle intervention investigated to the decision makers. 

This study reports that the intervention group had a higher cost in the “with study” scenario than in the “reference” scenario in all the simulations, which may suggest that people would have been better off without the intervention. The reason for this may be that the intervention group became better off in some risk factors, such as systolic blood pressure and BMI, but worse off in other risk factors, for example fasting plasma glucose, HbA1c and HDL, compared with their baseline risk factors (Table 1). Yet, the increase in cost for the intervention group was lower than for the control group. This may be a possible explanation for the lower estimation while the first-year risk factors remained same (sensitivity analysis 1d, Table 3) since in the first-year control group had more beneficial effects on some risk factors compared with the intervention group (Table 1). This may indicate that short-term lifestyle interventions are effective but that the adherence to healthy lifestyle habits is low, as suggested by others [52].

A cost-effectiveness analysis may adopt an optimistic assumption that the effect of the intervention will persist for life [53,54] and/or a pessimistic assumption that the effect of the intervention will persist only for the intervention period [31,55]. The assumption adopted drastically affects the results, as has been found in a recent review [10]. Salkeld et al. found that the cost-effectiveness ratio would be 20 times lower if the intervention effectiveness was assumed to persist 1 additional year beyond the intervention period [56]. On the other hand, if the intervention effectiveness is assumed to persist 1 year rather than for life, the cost-effectiveness ratio would be ten times higher [53]. Van Baal et al. found that if 100% of their participants maintained the intervention effect, the result would be three times lower than for the base case where 23% people maintained the effect [57]. There is no agreed-upon view on how long the effectiveness of the intervention persists. The sensitivity analysis of our study showed a cost saving with lifelong intervention effectiveness and a reduction in cost and in QALYs gained when we assumed lower/shorter persistence of the intervention. Finally, the intervention was no longer cost-saving when effectiveness was considered for only the intervention period.

The MIs predicted higher cost savings compared with the base case analysis (sensitivity analysis 2a). Carrying the last observation forward could not properly capture the magnitude of the risk factor reduction and the results may be biased. The MI method was able to capture the change to some extent and this explains the higher cost saving as well as the higher QALY gain. A previous cost-effectiveness analysis has shown that MIs provide lower costs per QALY [58], which is in line with our findings.

The present study only considered the benefits of lifestyle interventions related to MeSy and its associated diseases but no other potential beneficial outcomes on cancer incidence [22], pain reduction [23] or reduced mental suffering [24]. In this sense, the result of this cost-effectiveness analysis was underestimated. Moreover, we need to be cautious when using the term “metabolic syndrome (MeSy)”. This is still a controversial term: some researchers advocate the clinical usefulness of the term [59] while others disagree with this [60].

The modelling approach is an alternative approach to conducting a large RCT with a long follow-up. In many cases, RCTs do not contain all the variables required for model simulation. Consequently, researchers need to make some assumptions about the variables [34,50,54], which sometimes leads to biased results. The strength of the Björknäs intervention was that it included all the anthropometric and physiological characteristics of the participants required for the model simulation. Additionally, the participants came from primary health care and the intervention was provided in primary health care settings with limited resources.

A limitation of this study was that we did not consider out-of-pocket expenses for the participants who continued physical activity after the intervention terminated. Participants who continued exercising might also have gained QALYs and therefore the overall effect on the result may be negligible. Moreover, considering that clinical parameters of the intervention were few in a small number of people, the intervention effect might be overestimated or underestimated. Furthermore, participants might have been particularly motivated to participate in lifestyle interventions and therefore tried to adhere with that. In that sense, the effect of intervention might not represent the effectiveness achieved in a non-trial setting. An ideal lifestyle intervention for cost-effectiveness purpose needs to consider the sample size requirement for economic evaluation beside clinical efficacy and as well as a longer follow up to validate the result of model simulation with real world situation.

## Conclusion

The lifestyle intervention provided in the Swedish Björknäs intervention was cost-saving both in the short and in the long term. Financial constraints should not prevent the implementation of lifestyle interventions in primary health care settings.

## Supporting Information

File S1 **A model for economic evaluations of metabolic syndrome interventions - technical report (revised 2011).**
(DOCX)

## References

[1] Kastorini CM, Milionis HJ, Esposito K, Giugliano D, Goudevenos JA et al. (2011) The effect of mediterranean diet on metabolic syndrome and its components: A meta-analysis of 50 studies and 534,906 individuals. J Am Coll Cardiol 57: 1299–1313. doi:10.1016/j.jacc.2010.09.073. PubMed: 21392646. 21392646

[2] Churilla JR, Zoeller RF (2008) Physical Activity: Physical Activity and the Metabolic Syndrome: A Review of the Evidence. Am J Lifestyle Med 2: 118–125. doi:10.1177/1559827607311981.

[3] Horton ES (2009) Effects of Lifestyle Changes to Reduce Risks of Diabetes and Associated Cardiovascular Risks: Results from Large Scale Efficacy Trials. Obesity 17: S43–S48. doi:10.1038/oby.2009.388. PubMed: 19927146. 19927146

[4] Isomaa B, Almgren P, Tuomi T, Forsén B, Lahti K et al. (2001) Cardiovascular Morbidity and Mortality Associated With the Metabolic Syndrome. Diabetes Care 24: 683–689. doi:10.2337/diacare.24.4.683. PubMed: 11315831. 11315831

[5] Lorenzo C, Okoloise M, Williams K, Stern MP, Haffner SM (2003) The Metabolic Syndrome as Predictor of Type 2 Diabetes: The San Antonio Heart Study. Diabetes Care 26: 3153–3159. doi:10.2337/diacare.26.11.3153. PubMed: 14578254. 14578254

[6] Roger VL, Go AS, Lloyd-Jones DM, Adams RJ, Berry JD et al. (2011) Heart Disease and Stroke Statistics—2011. Update / 1 About 1. About These Statistics / 2 American Heart Association 's 2020 Impact Goals / 3. Cardiovascular Diseases / 4. Subclinical Atherosclerosis / 5. Coronary Heart Disease, Acute Coronary Syndrome, and Angina Pectoris / 6. Stroke (Cerebrovascular Disease) / 7. High Blood Pressure / 8. Congenital Cardiovascular Defects / 9. Cardiomyopathy and Heart Failure / 10. Other Cardiovascular Diseases / 11. Family History and Genetics / 12. Risk Factor: Smoking/Tobacco Use / 13. Risk Factor: High Blood Cholesterol and Other Lipids / 14. Risk Factor: Physical Inactivity / 15. Risk Factor: Overweight and Obesity / 16. Risk Factor: Diabetes Mellitus / 17. End-Stage Renal Disease and Chronic Kidney Disease / 18. Metabolic Syndrome / 19. Nutrition / 20. Quality of Care / 21. Medical Procedures / 22. Economic Cost of Cardiovascular Disease / 23. At-a-Glance Summary Tables / 24. Glossary. Circulation 123: e18–e209

[7] Grassi G, Seravalle G, Quarti-Trevano F, Dell’Oro R, Bombelli M et al. (2009) Metabolic syndrome and cardiometabolic risk: An update. Blood Press 18: 7–16. doi:10.1080/08037050802677695. PubMed: 19148840. 19148840

[8] Hollman G, Kristenson M (2008) The prevalence of the metabolic syndrome and its risk factors in a middle-aged Swedish population – Mainly a function of overweight? Eur J Cardiovasc Nurs 7: 21–26. doi:10.1016/j.ejcnurse.2008.01.039. PubMed: 17586094. 17586094

[9] Welin L, Adlerberth A, Caidahl K, Eriksson H, Hansson PO et al. (2008) Prevalence of cardiovascular risk factors and the metabolic syndrome in middle-aged men and women in Gothenburg, Sweden. BMC Public Health 8: 403. doi:10.1186/1471-2458-8-403. PubMed: 19063738. 19063738 PMC2621201

[10] Saha S, Gerdtham UG, Johansson P (2010) Economic Evaluation of Lifestyle Interventions for Prevention of Diabetes and Cardiovascular Diseases. Int J Environ Res Public Health 7: 3150–3159. doi:10.3390/ijerph7083150. PubMed: 20948954. 20948954 PMC2954575

[11] Eriksson KM, Westborg CJ, Eliasson MC (2006) A randomized trial of lifestyle intervention in primary healthcare for the modification of cardiovascular risk factors. Scand J Public Health 34: 453–461. doi:10.1080/14034940500489826. PubMed: 16990155. 16990155

[12] Eriksson MK, Franks PW, Eliasson M (2009) A 3-year randomized trial of lifestyle intervention for cardiovascular risk reduction in the primary care setting:The Swedish Bjorknas study. PLOS ONE 4: e5195. doi:10.1371/journal.pone.0005195. PubMed: 19365563. 19365563 PMC2664964

[13] Banta HD, de Wit GA (2008) Public Health Services and Cost-Effectiveness Analysis. Annu Rev Public Health 29: 383–397. doi:10.1146/annurev.publhealth.29.020907.090808. PubMed: 18173390. 18173390

[14] Griffin S, Rice N, Sculpher M (2009) Economic evaluation of public health interventions. In: Kelly A Evidence-based Public Health. Oxford: Oxford University Press pp. 111–127.

[15] the Swedish National Board of Health and Welfare National Guidelines for Methods of Preventing Disease – summary. Available: http://www.socialstyrelsen.se/nationalguidelines/nationalguidelinesformethodsofpreventingdisease. (Accessed 30.01.2013).

[16] Ashenfelter O, Card D (1985) Using the Longitudinal Structure of Earnings to Estimate the Effect of Training Programs. Rev Econ Statist 67: 648–660. doi:10.2307/1924810.

[17] Bassanini A, Venn D (2007) Assessing the impact of labour market policies on productivity: a difference-in-differences approach. OECD Publishing.

[18] Rodríguez-Oreggia E (2012) Hurricanes and labor market outcomes: Evidence for Mexico. Glob Environ Change 1: 351–359.

[19] Gilmer TP, Roze S, Valentine WJ, Emy-Albrecht K, Ray JA et al. (2007) Cost-Effectiveness of Diabetes Case Management for Low-Income Populations. Health Serv Res 42: 1943–1959. doi:10.1111/j.1475-6773.2007.00701.x. PubMed: 17850527. 17850527 PMC2254564

[20] Li G, Zhang P, Wang J, Gregg EW, Yang W et al. (2008) The long-term effect of lifestyle interventions to prevent diabetes in the China Da Qing Diabetes Prevention Study: a 20-year follow-up study. Lancet 371: 1783–1789. doi:10.1016/S0140-6736(08)60766-7. PubMed: 18502303. 18502303

[21] Artinian NT, Fletcher GF, Mozaffarian D, Kris-Etherton P, Van Horn L et al. (2010) Interventions to Promote Physical Activity and Dietary Lifestyle Changes for Cardiovascular Risk Factor Reduction in Adults: A Scientific Statement From the American Heart Association. Circulation 122: 406–441. doi:10.1161/CIR.0b013e3181e8edf1. PubMed: 20625115. 20625115 PMC6893884

[22] Brown CH, Baidas SM, Hajdenberg JJ, Kayaleh OR, Pennock GK et al. (2009) Lifestyle Interventions in the Prevention and Treatment of Cancer. Am J Lifestyle Med 3: 337–348. doi:10.1177/1559827609334983.

[23] Mattila R, Malmivaara A, Kastarinen M, Kivelä SL, Nissinen A (2004) Effects of lifestyle intervention on neck, shoulder, elbow and wrist symptoms. Scand J Work Environ Health 30: 191–198. doi:10.5271/sjweh.779. PubMed: 15250647. 15250647

[24] Walsh R (2011) Lifestyle and mental health. Am Psychol 66: 579–592. doi:10.1037/a0021769. PubMed: 21244124. 21244124

[25] Eriksson MK, Hagberg L, Lindholm L, Malmgren-Olsson EB, Osterlind J et al. (2010) Quality of life and cost-effectiveness of a 3-year trial of lifestyle intervention in primary health care. Arch Intern Med 170: 1470–1479. doi:10.1001/archinternmed.2010.301. PubMed: 20837834. 20837834

[26] Sculpher MJ, Claxton K, Drummond M, McCabe C (2006) Whither trial-based economic evaluation for health care decision making? Health Econ 15: 677–687. doi:10.1002/hec.1093. PubMed: 16491461. 16491461

[27] Li G, Zhang P, Wang J, Gregg EW, Yang W et al. (2008) The long-term effect of lifestyle interventions to prevent diabetes in the China Da Qing Diabetes Prevention Study: a 20-year follow-up study. Lancet 371: 1783–1789. doi:10.1016/S0140-6736(08)60766-7. PubMed: 18502303. 18502303

[28] Lindström J, Ilanne-Parikka P, Peltonen M, Aunola S, Eriksson JG et al. (2008) Sustained reduction in the incidence of type 2 diabetes by lifestyle intervention: follow-up of the Finnish Diabetes Prevention Study. Lancet 368: 1673–1679. PubMed: 17098085. 10.1016/S0140-6736(06)69701-8 17098085

[29] Galani C, Schneider H, Rutten FF (2007) Modelling the lifetime costs and health effects of lifestyle intervention in the prevention and treatment of obesity in Switzerland. Int J Public Health 52: 372–382. doi:10.1007/s00038-007-7014-9. PubMed: 18369000. 18369000

[30] Herman WH, Brandle M, Hicks K, Sorensen S, Zhang P, Hamman RF, Ackermann RT, Engelgau MM (2005) The cost-effectiveness of lifestyle modification or metformin in preventing type 2 diabetes in adults with impaired glucose tolerance. Ann Intern Med: 2005: 323–332 10.7326/0003-4819-142-5-200503010-00007 PMC2701392 15738451

[31] Jacobs-van der Bruggen MA, Bos G, Bemelmans WJ, Hoogenveen RT, Vijgen SM et al. (2007) Lifestyle interventions are cost-effective in people with different levels of diabetes risk: results from a modeling study. Diabetes Care 30: 128–134. doi:10.2337/dc06-0690. PubMed: 17192345. 17192345

[32] Palmer AJ, Roze S, Valentine WJ, Spinas GA, Shaw JE et al. (2004) Intensive lifestyle changes or metformin in patients with impaired glucose tolerance: Modeling the long-term health economic implications of the diabetes prevention program in Australia, France, Germany, Switzerland, and the United Kingdom. Clinical Therapeutics 26: 304–321. doi:10.1016/S0149-2918(04)90029-X. PubMed: 15038953. 15038953

[33] Eddy DM, Schlessinger L, Kahn R (2005) Clinical Outcomes and Cost-Effectiveness of Strategies for Managing People at High Risk for Diabetes. Ann Intern Med 143: 251–264. doi:10.7326/0003-4819-143-4-200508160-00006. PubMed: 16103469. 16103469

[34] Johansson P, Östenson C-G, Hilding AM, Andersson C, Rehnberg C et al. (2009) A cost-effectiveness analysis of a community-based diabetes prevention program in Sweden. Int J Technol Assess Health Care 25: 350–358. doi:10.1017/S0266462309990079. PubMed: 19619354. 19619354

[35] Feldman I, Hellström L, Jojhansson P (2013) Heterogeneity in cost-effectiveness of lifestyle counseling for metabolic syndrome risk groups-primary care patients in Sweden. Cost Effectiveness Res Allocation 11: 19 10.1186/1478-7547-11-19 PMC3765778 23984906

[36] Anderson KM, Odell PM, Wilson PW, Kannel WB (1991) Cardiovascular disease risk profiles. Am Heart J 121: 293–298 1985385 10.1016/0002-8703(91)90861-b

[37] Stern MP, Williams K, Haffner SM (2002) Identification of Persons at High Risk for Type 2 Diabetes Mellitus: Do We Need the Oral Glucose Tolerance Test? Ann Intern Med 136: 575–581. doi:10.7326/0003-4819-136-8-200204160-00006. PubMed: 11955025. 11955025

[38] Clarke PM, Gray AM, Briggs A, Farmer AJ, Fenn P et al. (2004) A model to estimate the lifetime health outcomes of patients with Type 2 diabetes: the United Kingdom Prospective Diabetes Study (UKPDS) Outcomes Model (UKPDS no. 68). Diabetologia 47: 1747–1759. doi:10.1007/s00125-004-1527-z. PubMed: 15517152. 15517152

[39] Feldman I, Johansson P, Lund C (2009) A model for economic evaluations of metabolic syndrome interventions Tech Rep

[40] Drummond MF, Sculpher MJ, Torrance GW, O'Brien BJ, Stoddart GL (2005) Methods for the Economic Evaluation of Health Care Programmes. Oxford: Oxford University Press.

[41] Statistics Sweden Consumer Price Index (CPI), Aavailable at http://www.scb.se/Pages/TableAndChart . Retrieved onpublished at whilst December year 1111 from ____272152.aspx. (Accessed 30.02.2013)

[42] Carlsson P, Anell A, Eliasson M (2006) Hälsoekonomi får allt större roll för sjukvårdens prioriteringar. Läkartidningen. 17153871

[43] Bank World GDP per capita. Avaialble at http://data.worldbank.org/indicator/NY.GDP.PCAP .CD, (Accessed on 30.02.2013).

[44] WHO (2005) CHOosing Intervention that are Cost Effective (WHO–CHOICE) World Health Organization. Available: http://www.who.int/choice/costs/CER_levels/en/index.html. (Accessed 14.11.2012).

[45] Wood AM, White IR, Thompson SG (2004) Are missing outcome data adequately handled? A review of published randomized controlled trials in major medical journals. Clin Trials 1: 368–376. doi:10.1191/1740774504cn032oa. PubMed: 16279275. 16279275

[46] Lavori PW, Dawson R, Shera D (1995) A multiple imputation strategy for clinical trials with truncation of patient data. Statist Med 14: 1913–1925. doi:10.1002/sim.4780141707. PubMed: 8532984. 8532984

[47] Rubin D (1987) Multiple Imputation for Nonresponse in Surveys. New York: Wiley & Sons.

[48] Löfroth E, Lindholm L, Wilhelmsen L, Rosén M (2006) Optimising health care within given budgets: Primary prevention of cardiovascular disease in different regions of Sweden. Health Policy 75: 214–229. doi:10.1016/j.healthpol.2005.03.008. PubMed: 16005539. 16005539

[49] DPP group (2003) Within-Trial Cost-Effectiveness of Lifestyle Intervention or Metformin for the Primary Prevention of Type 2 Diabetes. Diabetes Care 26: 2518–2523

[50] Lindgren P, Lindström J, Tuomilehto J, Uusitupa M, Peltonen M et al. (2007) Lifestyle intervention to prevent diabetes in men and women with impaired glucose tolerance is cost-effective. Int J Technol Assess Health Care 23: 177–183. PubMed: 17493303. 17493303 10.1017/S0266462307070286

[51] Hagberg LA, Lindholm L (2005) Is promotion of physical activity a wise use of societal resources? Issues of cost–effectiveness and equity in health. Scand J Med Sci Sports 15: 304–312. doi:10.1111/j.1600-0838.2004.00415.x. PubMed: 16181254. 16181254

[52] Fappa E, Yannakoulia M, Pitsavos C, Skoumas I, Valourdou S et al. (2008) Lifestyle intervention in the management of metabolic syndrome: could we improve adherence issues? Nutrition 24: 286–291. doi:10.1016/j.nut.2007.11.008. PubMed: 18201869. 18201869

[53] Finkelstein EA, Khavjou O, Will JC (2006) Cost-Effectiveness of WISEWOMAN, a Program Aimed at Reducing Heart Disease Risk among Low-Income Women. J Womens Health 15: 379–389. doi:10.1089/jwh.2006.15.379. PubMed: 16724886. 16724886

[54] Lindgren P, Fahlstadius P, Hellenius M-L, Jönsson B, de Faire U (2003) Cost-effectiveness of primary prevention of coronary heart disease through risk factor intervention in 60-year-old men from the county of Stockholm – a stochastic model of exercise and dietary advice. Prev Med 36: 403–409. doi:10.1016/S0091-7435(02)00060-9. PubMed: 12649048. 12649048

[55] Colagiuri S, Walker AE (2008) Using An Economic Model Of Diabetes To Evaluate Prevention And Care Strategies In Australia. Health Aff 27: 256–268. doi:10.1377/hlthaff.27.1.256. PubMed: 18180502. 18180502

[56] Salkeld G, Phongsavan P, Oldenburg B, Johannesson M, Convery P et al. (1997) The cost-effectiveness of a cardiovascular risk reduction program in general practice. Health Policy 41: 105–119. doi:10.1016/S0168-8510(97)00015-8. PubMed: 10169297. 10169297

[57] Van Baal PH, Van den Berg M, Hoogenveen RT, Vijgen SM, Engelfriet PM (2008) Cost-effectiveness of a low-calorie diet and orlistat for obese persons: modeling long-term health gains through prevention of obesity-related chronic diseases. Value Health 11: 1033–1040. doi:10.1111/j.1524-4733.2008.00328.x. PubMed: 18494748. 18494748

[58] Wallerstedt SM, Bladh L, Ramsberg J (2012) A cost-effectiveness analysis of an in-hospital clinical pharmacist service. BMJ Open 2: 1. 10.1136/bmjopen-2011-000329 PMC3253415 22223840

[59] Grundy SM (2007) Metabolic syndrome: A multiplex cardiovascular risk factor. J Clin Endocrinol Metab 92: 399–404. PubMed: 17284640. 17284640 10.1210/jc.2006-0513

[60] Kahn R, Buse J, Ferrannini E, Stern M (2005) The Metabolic Syndrome: Time for a Critical Appraisal: Joint statement from the American Diabetes Association and the European Association for the Study of Diabetes. Diabetes Care 28: 2289–2304. doi:10.2337/diacare.28.9.2289. PubMed: 16123508. 16123508

