# Supplementary material for: Are Lifestyle Interventions in Primary Care Cost-Effective? – An Analysis Based on a Markov Model, Differences-In-Differences Approach and the Swedish Björknäs Study
Source: PLoS One. 2013 Nov 14;8(11):e80672. doi: 10.1371/journal.pone.0080672 (PMC3828270; doi:10.1371/journal.pone.0080672)
Supplement: File S1 — A model for economic evaluations of metabolic syndrome interventions - technical report (revised 2011). (DOCX) [file pone.0080672.s001.docx]

**A model for economic evaluations of metabolic syndrome interventions**

**- technical report (revised 2011)**

Inna Feldman

Caroline Lund

Karoline Jeppsson

Pia Johansson

A model for economic evaluations of metabolic syndrome interventions.

Technical report (revised 2011)

Year 2011.

Authors:

Inna Feldman

Uppsala University

Sweden

Pia Johansson

Caroline Lund Karoline Jeppsson (revised version)

Karolinska Institutet

Sweden

**The report is available at:**

<http://www.folkhalsoguiden.se/upload/Folksjukdomar/Rapporter/metabolic%20cea%20model%20technical%20report%20-revised%20version%202011.pdf>

or from the authors:

inna.feldman@kbh.uu.se

pia.johansson.fullersta@gmail.com

Content

[1 Introduction 5](#_Toc237342024)

[2 Method 6](#_Toc237342025)

[2.1 The metabolic syndrome 6](#_Toc237342026)

[2.1 Diseases 6](#_Toc237342027)

[2.2 The model 8](#_Toc237342028)

[2.3 Risk factors in model 10](#_Toc237342029)

[3 Material 12](#_Toc237342030)

[3.1 The risks 12](#_Toc237342031)

[3.1.1 Disease risks 12](#_Toc237342032)

[3.1.2 Death risks 15](#_Toc237342033)

[3.2 The costs 18](#_Toc237342034)

[3.2.1 Medical treatment cost 18](#_Toc237342035)

[3.2.2 Other societal costs 21](#_Toc237342036)

[3.3 The health effects 24](#_Toc237342037)

[3.4 Sensitivity analyses 26](#_Toc237342038)

[3.4.1 Univariate analyses 26](#_Toc237342039)

[3.4.2 Multivariate analysis 27](#_Toc237342040)

[3.4.3 Methodological aspects 27](#_Toc237342041)

[3.4.4 Probabilistic sensitivity analysis 28](#_Toc237342042)

[4. Model output 29](#_Toc237342043)

[4.1 Model estimates from risk factor levels 29](#_Toc237342044)

[4.2 Detailed model output 31](#_Toc237342045)

[4.4 Sensitivity analyses 33](#_Toc237342046)

[5 Discussion 36](#_Toc237342047)

[5.1 The structure of the model 36](#_Toc237342048)

[5.2 The inputs of the model 38](#_Toc237342049)

[5.2.1 The risks 38](#_Toc237342050)

[5.2.2 The costs 41](#_Toc237342051)

[5.2.3 The health effects 43](#_Toc237342052)

[5.3 The results of the model 45](#_Toc237342053)

[5.4 The value of the model to the decision-maker 48](#_Toc237342054)

[6 References 49](#_Toc237342055)

Tables

[Table 1. Risk factor levels for metabolic syndrome. 6](#_Toc238026962)

[Table 2. The model diseases, with ICD-10 codes. 7](#_Toc238026963)

[Table 3. Diabetes-related complications in Sweden. 8](#_Toc238026964)

[Table 4. Variables included in the model. 10](#_Toc238026966)

[Table 5. Risk factor levels for report simulations. 11](#_Toc238026967)

[Table 6. Transition probabilities in the model. 12](#_Toc238026968)

[Table 7. The annual risks of CHD 12](#_Toc238026969)

[Table 8. Distribution of CHD 14](#_Toc238026970)

[Table 9. The annual risks of stroke 14](#_Toc238026971)

[Table 10. The annual risks of diabetes 14](#_Toc238026972)

[Table 11. The annual risks of macro complications to diabetes 14](#_Toc238026973)

[Table 12. The annual risks of micro complications to diabetes 15](#_Toc238026974)

[Table 13. The average death risks, all causes. 16](#_Toc238026975)

[Table 14. The unrelated death risks. 16](#_Toc238026976)

[Table 15. The death risk in AMI during the first year. 17](#_Toc238026977)

[Table 16. The death risk in stroke during the first year. 17](#_Toc238026978)

[Table 17. The death risk in CHF. 17](#_Toc238026979)

[Table 18. The death risk in IHD. 18](#_Toc238026980)

[Table 19. The death risk in macro complications to diabetes. 18](#_Toc238026981)

[Table 20. Standard costs for primary care. 19](#_Toc238026982)

[Table 21. Medical treatment cost. 20](#_Toc238026983)

[Table 22. Pharmaceutical costs. 21](#_Toc238026984)

[Table 23. Costs for community care and technical aids. 22](#_Toc238026985)

[Table 24. Costs for informal care and other costs for patients and relatives 23](#_Toc238026986)

[Table 25. Productivity costs. 23](#_Toc238026987)

[Table 26. Average Swedish population quality-of-life weights. 25](#_Toc238026988)

[Table 27. Disease-specific quality of weight losses. 25](#_Toc238026989)

[Table 28. Model estimates from selected risk factor levels. 30](#_Toc238026990)

[Table 29. Estimated disease costs per cost item, men aged 50 years. 31](#_Toc238026991)

[Table 30. Estimated disease costs per cost item, women aged 50 years. 32](#_Toc238026992)

[Table 31. Costs per Swedish payer. 33](#_Toc238026993)

[Table 32. Comparison of model estimated outcomes with other models. 47](#_Toc238026998)

Figures

[Figure 1. State-transition diagram 9](#_Toc237342122)

[Figure 2. Estimated incidence in metabolic diseases 32](#_Toc237342151)

[Figure 3. Estimated mortality 33](#_Toc237342152)

[Figure 4. Sensitivity analyses on model parameters. 34](#_Toc237342153)

[Figure 5. Bootstraps from model simulations 35](#_Toc237342154)

# 1 Introduction

The metabolic syndrome model is constructed to enable cost-effectiveness analyses on interventions against the syndrome. The model aims to follow international and Swedish recommendations on how to perform cost-effectiveness analyses in health and medicine. The aim of this report is to detail the model data and assumptions, to report some model results, and to discuss the model validity.

The model incorporates the main diseases due to the metabolic syndrome; cardiovascular disease (CVD), including stroke, and diabetes mellitus type 2, including diabetes-related complications. The model simulates the societal costs and health effects of different levels of the metabolic syndrome risk factors blood pressure, cholesterol level, glucose level and BMI (body mass index). Furthermore, the risk factor glycated haemoglobin (HbA1c) is used to assess the risk of contracting diabetes-related complications. The disease-related costs seek to include costs to all sectors of society, such as medical treatment cost, costs for institutional care, pharmaceuticals, for informal care and other costs for patients and relatives, and productivity costs due to morbidity. The health effects are measured as QALYs (quality-adjusted life-years) and lost life-years (YLS).

The model is constructed for cost-effectiveness analyses of interventions that affect the risk factors for the syndrome. When interventions manage to change risk factor levels, the model can be used to calculate ensuing changes in costs and health. The purpose of the model is thus to value the consequences of a health care programme, as termed by Drummond and co-authors (2005). These consequences in terms of disease-related costs and health effects might then be inserted into the cost-effectiveness ratio, together with the costs of implementing the programme, to calculate the costs per health effect.

The present report is a revised version of the technical report on the model published in 2009. The revision only considers the diabetes and diabetes-related diseases, and the only changes implemented concerns the risk functions for diabetes-related complications.

2 Method

2.1 The metabolic syndrome

The metabolic syndrome is a condition characterized by a combination of biological risk factors that are related to lifestyles such as physical activity and food habits. Since the 1920s there have been a number of clinical criterias for the syndrome. The most recent definition is from the International Diabetes Federation (Alberti et al, 2006), where the criteria for metabolic syndrom is central obesity, measured as waist circumference, plus any two of four additional risk factors, as defined in table 1.

The metabolic syndrome increases the risks for cardiovascular disease (CVD) and type 2 diabetes, as well as all-cause mortality. The relative risks, using an older but similar definition of metabolic syndrome, has been summarized as 1.27 (95% confidence interval 0.90-1.78) for all-cause mortality, 1.65 (1.38-1.99) for CVD, and 2.99 (1.96-4.57) for diabetes (Ford, 2005).

2.1 Diseases

The model is inspired by a previously published US model on the metabolic syndrome (Caro et al, 2007). The model reported here incorporates the most common diseases due to the metabolic syndrome; cardiovascular disease (CVD) and type 2 diabetes, see table 2. In the model, CVD includes coronary heart disease (CHD) and stroke. CHD is divided into four disease groups: Acute myocardial infarction (AMI), Ischemic heart disease (IHD), Congestive heart failure (CHF) and Sudden death. IHD includes all heart disease except AMI, CHF and sudden death. Diabetes type 2 diabetes is included, as well as common complications to diabetes, but not diabetes type 1 (ICD-10 code E10). The diabetes-related complications are

Table 1. Risk factor levels for metabolic syndrome, according to IDF.

| **Definition** | **Men** | **Women** |
| --- | --- | --- |
| Waist circumference | ≥ 94 cm* | ≥ 80 cm* |
| Triglycerides | ≥ 1.7 mmol/l | ≥ 1.7 mmol/l |
| HDL- cholesterol | < 1.03 mmol/l | < 1.29 mmol/l |
| Systolic blood pressure | ≥ 130 mmHg | ≥ 130 mmHg |
| Fasting plasma glucose | ≥ 5.6 mmol/l | ≥ 5.6 mmol/l |

* for Europids

Source: Alberti et al, 2006.

Table 2.. The model diseases, with ICD-10 codes.

| **Disease** | **ICD-10** |
| --- | --- |
| *Cardiovascular disease, CVD* |  |
| Coronary heart disease, CHD |  |
| Acute myocardial infarction, AMI | I21 I22 I23 |
| Ischemic heart disease, IHD | I20 I24 I25 |
| Congestive heart failure, CHF | I50 |
| Sudden death | I46.1 |
| Stroke | I61 I63 I64 |
|  |  |
| Diabetes type 2 | E11.0 E11.1 E11.8 E11.9 |
| *Diabetes-related complications:* |  |
| Macrovascular |  |
| Acute myocardial infarction, AMI | I21 I22 I23 |
| Ischemic heart disease, IHD | I20 I24 I25 |
| Congestive heart failure, CHF | I50 |
| Sudden death | I46.1 |
| Stroke | I61 I63 I64 |
| Peripheral vascular complication | E11.5 I79.2 |
| Microvascular |  |
| Renal failure (nephropathy) | E11.2 N18 N19 Z49 |
| Retinopathy and blindness | E11.3 H28.0 H36.0 H54 |
| Neuropathy | E11.4 G73.0 G99.0 G59.0 G63.2 |
| Foot ulcers and amputation | E11.6 L97 L98 M14.2 M14.6 M90.8 |
| Both complications |  |
| Multiple complications | E11.7 |

divided into macrovascular and microvascular complications. The macro complications include CHD, stroke and peripheral vascular disease, while the micro complications include nephropathy (renal failure), retinopathy (decreased eye-sight), neuropathy and foot ulcers. The definition and division of diabetes-related complications are taken from the CODE-2 study (Williams et al, 2002). It is possible to suffer from both macro and micro complications at the same time.

Table 3. Diabetes-related complications in Sweden, prevalence and six month incidence.

|  | **Prevalence, %** | **Incidence, %** |
| --- | --- | --- |
| Blindness | 3.7 | 0.1 |
| Dialysis | 0.5 | 0.0 |
| Foot ulcer | 5.4 | 1.8 |
| Amputation | 1.3 | 0.1 |
| Myocardial infarction | 9.4 | 0.6 |
| Angina | 17.2 | 0.9 |
| Coronary artery bypass graft | 4.0 | 0.0 |
| Stroke | 6.8 | 1.2 |

Source: Henriksson et al, 2000.

The most common diabetes-related complications in Sweden are the macrovascular, see table 3, in particular angina pectoris and acute myocardial infarction, while the microvascular are considerably less frequent, according to the Swedish part of the study CODE-2 (Henriksson et al, 2000).

2.2 The model

The model is a partly stochastic Markov-cycle tree, constructed to enable Monte Carlo simulations. A Markov model is a health state transition model that uses probabilities for transitions between health states (Sonnenberg & Beck, 1993; Briggs & Schulpher, 1998; Kuntz & Weinstein, 2001). These probabilities are the risks of contracting CHD, stroke, diabetes and diabetes-related complications given the risk factor levels, as well as the risks of death from the diseases or from other causes.

The starting point in the model is At risk, see figure 1. Individuals might transition from At risk to any of the health states Diabetes, the CHD diseases or Stroke. CHD is further divided into four disease groups, Acute myocardial infarction (AMI), Congestive heart failure (CHF), Ischemic heart disease (IHD) and Sudden death. Individuals that have transitioned to Diabetes run risks of contracting complications, which is modelled as Diabetes without complications, Micro complications, Macro complications and Both complications. The health states are mutually exclusive and collectively exhaustive. Transitions between the CVD health states and Diabetes are not allowed, but the CVD diseases are included in the Macro complications. Individuals in the Macro complications thus suffers from both diabetes and CVD.

Figure 1. State-transition diagram

Stroke

Diabetes

At risk

CHD

Dead

Dead other

IHD

AMI

CHF

Both compl

Micro compl

Macro compl

Sudden death

In all health states except Diabetes, the individuals also run the risks of death, either in the respective diseases, Dead, or in unrelated causes, Dead other. For individuals in Diabetes without complications the only risk of death is from unrelated causes, because epi-demiological data indicates that the excess death risks for diabetes patients are due to complications (Clarke et al, 2004; Malmberg et al, 1999). In the health state At risk, only unrelated death is possible.The model is created in DATA Pro Health Care (Treeage Inc, 2007). The Markov stages are one year long, without half cycle correction. The model covers adults, with the termination age set at 85 years. Incremental costs and health effects are accumulated during the time spent in each health state. In addition, some medical treatment costs and health effects are incurred during transitions between health states, such as during the acute phase of a myocardial infarction.

The model is run as a Monte Carlo simulation with 10,000 repetitions. The simulations result in the expected value with accompanying distributions for the

Table 4. Variables included in the model.

| **Abbreviations** | **Definitions/values** |
| --- | --- |
| Age | Age in years |
| Sex | 1 for female; 0 for male |
| Smok | 1 for current smoker, 0 otherwise |
| BMI | Body mass index (m/kg^2^ ) |
| FPG | Fasting plasma glucose (mmol/l) |
| HbA1c | Glycated haemoglobin (%) |
| Chol | Total cholesterol (mmol/l) |
| HDL | HDL – cholesterol (mmol/l) |
| SBP | Systolic blood pressure (mmHg) |
| Diabetes | 1 if has; 0 otherwise |
| YD | Years with diabetes |

defined risk factor values. The costs and health effects for different risk factor values are then compared outside the model.

2.3 Risk factors in model

The risk factors included in the model are dependent on the risk functions that are available to estimate the disease risks. Fortunately, all but two of the metabolic syndrome risk factors, as defined in table 1, have been included in well-known risk functions for diabetes type 2 and CVD. The risk factor waist circumference was not possible to include in the model, why it is replaced by body mass index (BMI). BMI was included in a previous metabolic syndrome definition, by the WHO, with a risk level of higher than 30 (Alberti et al, 2006) and have been frequently used in epidemiological studies on the syndrome (Ford, 2005). The risk factor triglycerides was not possible to include in the model. Furthermore, the risk functions include a coefficient for the total cholesterol level. Two different risk factors were used to estimate the risks of diabetes and the diabetes-related complications. The metabolic syndrome risk factor fasting plasma glucose (FPG) was employed to calculate the risk of contracting diabetes (WHO, 2006), while the risk factor glycated haemoglobin (HbA1c), that measures the average glucose levels, was used to estimate the risks for diabetes-related complications. To avoid contracting diabetes-related complications HbA1c levels below 6 % are recommended by Swedish guidelines (National Board of Health and Welfare, 2010). The risk factors included in the model are detailed in table 4.

The risk factor levels used for simulations for specific interventions are naturally defined by the patients included in the interventions. For this report, some risk factor levels have been chosen selectively to illustrate the model results, see table 5. The increased risk levels are the threshold values either for the IDF metabolic syndrome definition (see also table 1) or for diabetes-related complications as defined by national guidelines. The high values seek to represent a fairly ill population while the low values represent a population with a low risk for the metabolic syndrome diseases.

Table 5. Risk factor levels for report simulations.

| **Risk factors** | **Low** | **Increased risk** | **High** |
| --- | --- | --- | --- |
| BMI | 22 | 25 | 30 |
| HDL-cholesterol | 1.5 | 1.3 (women)  1.0 (men) | 0.8 |
| Systolic blood pressure | 120 | 130 | 145 |
| Fasting plasma glucose | 4.2 | 5.6 | 6.1 |
| Cholesterol | 4 | 5 | 6.5 |
| HbA1c | 4.2 | 6 | 7 |

The model results in this report include one simulation with all risk factors at high values, and one with all risk factors at low values. Furthermore, to demonstrate the effects of each risk factor, one simulation each is run with respective risk factor set at a high value and the others at low values. Note that the values at the increased risk levels are not used in any simulations. Every set of risk factor values is run for men and women separately and with two starting ages, 35 years and 50 years.

3 Material

The model aims to comply with recommendations for cost-effectiveness analyses (Gold et al, 1996; Drummond et al, 2005) and in particular with the Swedish recommendations for pharmaceutical cost-effectiveness analyses (LFN, 2003). The model reflects the societal perspective and thus aims to include all metabolic syndrome-related costs for all sectors of society.

All cost data besides medical treatment cost are taken from Swedish previously published studies, as well as the average quality-of life weights. All but one of the death risks are taken from Swedish databases, while the disease risks and the disease-related quality-of-life weights are taken from international studies.

All cost are measured in year 2004 SEK (1 Euro=9.13 SEK), converted by the Swedish con-sumer price index (from SCB) and the official Swedish exchange rate (from The Riksbank) if necessary. The annual discount rate is 3% for both costs and effects.

3.1 The risks

The structure of the model risks are similar to the Caro et al (2007) model, see table 6 for a summary of the transition probabilities including references to the Markov-tree, names, and computation method.

3.1.1 Disease risks

The disease risk estimates are based on the same studies as the Caro et al (2007) model, i.e. the Framingham CVD risk function (Anderson et al, 1991), a US study (San Antonio) on the risk of diabetes (Stern et al, 2002), and the British UKPDS (Clarke et al, 2004) on the risks of diabetes-related complications. The computations of transition probabilities from the risk functions are however somewhat different, see tables 7 to 12. Furthermore, the division of CHD events into particular diseases, necessary as the Framingham CHD risk function only calculates CHD events, are based on recent Swedish register data, see table 8.

Table 7. The annual risks of CHD

****15.5305+28.4441*Sex-1.479*Ln(Age)-14.4588*Ln(Age)*Sex+1.8515*(Ln(Age))^2^*Sex-0.9119*Ln(SBP)-0.2767*Smok-0.7181*Ln(Chol/HDL)-0.1759*Diabetes-0.1999*Diabetes*Sex

$P_{chd}$=1-Exp(-Exp((- **$\mu_{chd}$**)/ Exp(0.9145-0.2784*****)))

Source: Caro et al, 2007; Anderson et al, 1991

Table 6. Transition probabilities in the model.

| **Node** | **Branch** | **Name** | **Computation** | **Reference** |
| --- | --- | --- | --- | --- |
| At risk | CHD | $P_{chd}$ | Table 7 | Caro et al, 2007; Anderson et al, 1991 |
|  | Stroke | $P_{str}$ | Table 9 | Caro et al, 2007; Anderson et al, 1991 |
|  | Diabetes | $P_{diab}$ | Table 10 | Caro et al, 2007; Stern et al, 2002 |
|  | Dead other | $P_{died}$ | Table 14 | The Swedish Cause of Death Register |
| CHD | AMI | $P_{chd\_ami}$ | Table 8 | Swedish statistics on diagnoses in in-patient care from the Hospital Discharge Register |
|  | IHD | $P_{chd\_ihd}$ | Table 8 |  |
|  | CHF | $P_{chd\_chf}$ | Table 8 |  |
|  | Sudden death | $P_{chd\_sd}$ | Table 8 |  |
| AMI | Dead AMI | $P_{died\_AMI}$ | 1st year - Table 15 2nd etc – Table 13 | The Swedish AMI Statistics; The Swedish Cause of Death Register |
|  | Dead other | $P_{died\_not\_AMI}$ | Table 14 | The Swedish Cause of Death Register |
| IHD | Dead IHD | $P_{died\_IHD}$ | Table 18 | Cooper et al, 2002 |
|  | Dead other | $P_{died\_not\_IHD}$ | Table 14 | The Swedish Cause of Death Register |
| CHF | Dead CHF | $P_{died\_CHF}$ | Table 17 | The Swedish Heart Failure Register |
|  | Dead other | $P_{died\_not\_CHF}$ | Table 14 | The Swedish Cause of Death Register |
| Stroke | Dead Stroke | $P_{died\_str}$ | 1st year - Table 16 2nd etc – Table 13 | The Swedish Stroke Register; The Swedish Cause of Death Register |
|  | Dead other | $P_{died\_not\_str}$ | Table 14 | The Swedish Cause of Death Register |
| Diabetes | Macro compl. | $P_{macro}$ | Table 11 | Caro et al, 2007; Clarke et al, 2004 |
|  | Micro compl. | $P_{micro}$ | Table 12 | Caro et al, 2007; Clarke et al, 2004 |
|  | Dead other | $P_{died}$ | Table 13 | The Swedish Cause of Death Register |
| Macro compl. | Both compl. | $P_{micro}$ | Table 12 | Clarke et al, 2004 |
|  | Dead macro compl. | $P_{died\_macro}$ | Table 19 | The Swedish Cause of Death Register |
|  | Dead other | $P_{died\_not\_macro}$ | Table 14 | The Swedish Cause of Death Register |
| Micro compl. | Both compl. | $P_{macro}$ | Table 11 | Clarke et al, 2004 |
|  | Dead micro compl. | 0 |  |  |
|  | Dead other | $P_{died}$ | Table 13 | The Swedish Cause of Death Register |
| Both compl. | Dead both compl. | $P_{macro}$ | Table 11 | The Swedish Cause of Death Register |
|  | Dead other | $P_{died\_not\_macro}$ | Table 14 | The Swedish Cause of Death Register |

Table 8. Distribution of CHD

|  | **< 65 years** | | **>65 years** | |
| --- | --- | --- | --- | --- |
|  | **Men** | **Women** | **Men** | **Women** |
| $P_{chd\_ami}$ | 0.41 | 0.32 | 0.37 | 0.37 |
| $P_{chd\_ihd}$ | 0.46 | 0.52 | 0.28 | 0.25 |
| $P_{chd\_chf}$ | 0.12 | 0.14 | 0.34 | 0.38 |
| $P_{chd\_sd}$ | 0.01 | 0.02 | 0.01 | 0.01 |

Source: Statistics on diagnoses in inpatient care from the Hospital Discharge Register, National Board of Health and Welfare, Centre for Epidemiology 2005

Table 9. The annual risks of stroke

****26.5116+0.2019*Sex-2.3741*Ln(Age)-2.4643*Ln(SBP)-0.3914*Smok-0.0229*Ln(Chol/HDL)-

0.3087*Diabetes-0.2627*Diabetes*Sex

$P_{str}$= 1-Exp(-Exp((- **$\mu_{str}$**)/ Exp(-0.04312*****)))

Source: Caro et al, 2007; Anderson et al, 1991

Table 10. The annual risks of diabetes

**$\mu_{diab}$**=-13.415+0.028*Age+0.661*Sex+1.422*FG+0.018*SBP-1.5093*HDL+0.070*BMI

$P_{diab}$=(1/(1+Exp(-**$\mu_{diab}$**)))/7.5

Source: Caro et al, 2007; Stern et al, 2002

Table 11. The annual risks of macro complications to diabetes

$\pi_{ami}$=Exp(7.06+0.055*Age+0.826*Sex+0.346*Smok+0.118*HbA1c+0.0101*SBP+1.19*Ln(Chol/HDL))

$h_{ami}$=$\pi_{ami}$ *0.006895*1.257*(YD)^0.257^

$\pi_{chf}$=Exp(-8.15+0.093*Age+0.066*BMI+0.157* HbA1c +0.0114*SBP)

$h_{chf}$=$\pi_{chf}$*0.000329*1.711*(YD) ^0.711^

$\pi_{ihd}$=Exp(-6.32+0.031*Age-0.471*Sex+0.125* HbA1c +0.0098*SBP+1.498*Ln(Chol /HDL))

$h_{ihd}$=$\pi_{ihd}$*0.004942*1.15*(YD)^0.15^

$\pi_{str}$=Exp(-9.7+0.085*Age-0.516*Sex+0.355*Smok+0.128* HbA1c +0.0276*SBP+0.113*( Chol /HDL))

$h_{str}$=$\pi_{str}$*0.000775*1.497*(YD)^0.497^

$P_{macro}$=1-Exp(-($h_{ami}$+$h_{chf}$+$h_{ihd}$+$h_{str}$))

Source: Caro et al, 2007; Clarke et al, 2004

Table 12. The annual risks of micro complications to diabetes

$\pi_{amp}$=Exp(-8.095+0.435* HbA1c +0.0228*SBP)

$h_{amp}$=$\pi_{amp}$i*0.000164*( YD)^0.451^*1.451

$\pi_{bliind}$=Exp(-6.54+0.069*Age+0.221* HbA1c)
=$\pi_{bliind}$*0.001559*( YD)^0.154^*1.154

$\pi_{renal}$=Exp(-9.75+0.0404*SBP)

=$\pi_{renal}$*0.0004*( YD)^0.865^*1.865

$P_{micro}$=1-Exp(-($h_{amp}$++))

Source: Caro et al, 2007, Clarke et al, 2004

### 3.1.2 Death risks

The death risks are derived from Swedish registers; The Swedish Cause of Death Register, The Swedish AMI Statistics, The Swedish National Stroke Register, and The Swedish National Heart Failure Register, while the death risk in IHD is taken from a UK model (Cooper et al, 2002). The other disease-specific death risks are calculated from the causes of death in the available prevalence-based registers. All death risks are age group and gender-specific, see tables 13 to 19. Note that there is no excess risk of death from diabetes without complications, based on epi-demiological data (Clarke et al, 2004) and from micro complications, due to lack of data. For stroke and AMI the model only incorporates an excess risk of death during the first year, after which the death risk is assumed similar as the average death risk.

As CHD and stroke are common causes of death in Sweden (Swedish National Board of Health and Welfare, 2009) the risk for Dead other is the average all-cause mortality adjusted by the disease-specific death risk, age and gender-specific, in these health states, see table 14. These adjusted risks of unrelated death for the health states AMI, IHD, Stroke, CHF, and Macro complications are calculated by excluding cases of death related to AMI, IHD, stroke, CHF, and macro complications to diabetes, respectively, from the all-cause age group and gender-specific mortality.

Table 13. The average death risks, all causes.

|  | **$P_{died}$** | |
| --- | --- | --- |
| **age** | **Men** | **Women** |
| 35 | 0.001 | 0.001 |
| 40 | 0.001 | 0.001 |
| 45 | 0.002 | 0.002 |
| 50 | 0.004 | 0.002 |
| 55 | 0.006 | 0.004 |
| 60 | 0.010 | 0.006 |
| 65 | 0.017 | 0.010 |
| 70 | 0.029 | 0.017 |
| 75 | 0.047 | 0.030 |
| 80 | 0.086 | 0.056 |
| 85 | 0.184 | 0.150 |

Source: The Swedish Cause of Death Register, National Board of Health and Welfare, Centre for Epidemiology 2004.

Table 14. The unrelated death risks. Adjusted for deaths in AMI, IHD, stroke, CHF, and macro complications to diabetes.

|  | $P_{died\_not\_AMI}$ | | $P_{died\_not\_IHD}$ | | $P_{died\_not\_str}$ | | $P_{died\_not\_CHF}$ | | $P_{died\_not\_macro}$ | |
| --- | --- | --- | --- | --- | --- | --- | --- | --- | --- | --- |
| **age** | **Men** | **Women** | **Men** | **Women** | **Men** | **Women** | **Men** | **Women** | **Men** | **Women** |
| 30 | 0.00066 | 0.00038 | 0.00066 | 0.00038 | 0.00067 | 0.00037 | 0.00067 | 0.00037 | 0.00065 | 0.00037 |
| 35 | 0.00087 | 0.00059 | 0.00087 | 0.00059 | 0.00089 | 0.00059 | 0.00089 | 0.00059 | 0.00083 | 0.00058 |
| 40 | 0.00137 | 0.00093 | 0.00140 | 0.00093 | 0.00143 | 0.00092 | 0.00146 | 0.00094 | 0.00128 | 0.00089 |
| 45 | 0.00225 | 0.00152 | 0.00232 | 0.00157 | 0.00236 | 0.00155 | 0.00243 | 0.00159 | 0.00205 | 0.00146 |
| 50 | 0.00352 | 0.00243 | 0.00371 | 0.00246 | 0.00382 | 0.00242 | 0.00392 | 0.00249 | 0.00319 | 0.00231 |
| 55 | 0.00553 | 0.00394 | 0.00569 | 0.00401 | 0.00596 | 0.00397 | 0.00619 | 0.00408 | 0.00473 | 0.00374 |
| 60 | 0.00865 | 0.00615 | 0.00881 | 0.00623 | 0.00935 | 0.00629 | 0.00966 | 0.00643 | 0.00734 | 0.00575 |
| 65 | 0.01461 | 0.00904 | 0.01550 | 0.00917 | 0.01614 | 0.00927 | 0.01676 | 0.00965 | 0.01238 | 0.00802 |
| 70 | 0.02509 | 0.01526 | 0.02583 | 0.01592 | 0.02717 | 0.01598 | 0.02831 | 0.01667 | 0.02071 | 0.01311 |
| 75 | 0.04142 | 0.02627 | 0.04262 | 0.02757 | 0.04431 | 0.02729 | 0.04667 | 0.02897 | 0.03258 | 0.02152 |
| 80 | 0.07525 | 0.05027 | 0.07799 | 0.05168 | 0.08039 | 0.05111 | 0.08237 | 0.05364 | 0.05730 | 0.03764 |
| 85+ | 0.16400 | 0.13750 | 0.16343 | 0.13333 | 0.17272 | 0.13695 | 0.17131 | 0.13905 | 0.11837 | 0.09579 |

Source: The Swedish Cause of Death Register, National Board of Health and Welfare, Centre for Epidemiology, 2004.

Table 15. The death risk in AMI during the first year.

|  | $P_{died\_AMI}$, 1st year | |
| --- | --- | --- |
| **age** | **Men** | **Women** |
| 20 | 0.148 | 0.179 |
| 50 | 0.174 | 0.148 |
| 55 | 0.185 | 0.169 |
| 60 | 0.224 | 0.237 |
| 65 | 0.307 | 0.26 |
| 70 | 0.349 | 0.336 |
| 75 | 0.451 | 0.414 |
| 80 | 0.533 | 0.486 |
| 85 | 0.657 | 0.631 |

Source: The Swedish AMI Statistics, National Board of Health and Welfare, Centre for Epidemiology, 2005.

Table 16. The death risk in stroke during the first year.

|  | $P_{died\_str}$ 1st year | |
| --- | --- | --- |
| **age** | **Men** | **Women** |
| >64 | 0.06 | 0.05 |
| 65 | 0.09 | 0.08 |
| 75 | 0.16 | 0.18 |

Source: The Swedish National Stroke Register, 2006.

Table 17. The death risk in CHF.

|  | $P_{died\_CHF}$ | |
| --- | --- | --- |
| **age** | **Men** | **Women** |
| 50 | 0.061 | 0.015 |
| 65 | 0.224 | 0.133 |
| 85 | 0.330 | 0.269 |

Source: The Swedish National Heart Failure Register, 2006.

Table 18. The death risk in IHD.

$P_{died\_IHD}$=Exp(0.0458*Age-6.574)

Source: Cooper et al, 2002

Table 19. The death risk in macro complications to diabetes.

$P_{died\_m1}$=$P_{died\_AMI}$+$P_{died\_IHD}$-$P_{died\_AMI}$*$P_{died\_IHD}$

$P_{died\_m2}$=$P_{died\_str}$+$P_{died\_CHF}$-$P_{died\_str}$*$P_{died\_CHF}$

$P_{died\_macro}$=$P_{died\_m1}$+$P_{died\_m2}$-$P_{died\_m1}$*$P_{died\_m2}$

Source: The Swedish Cause of Death Register, National Board of Health and Welfare, Centre for Epidemiology, 2004.

## 3.2 The costs

All disease-related costs for all parts of society are included, if available, thus reflecting the societal perspective. The medical treatment costs are taken from the Stockholm County Council healthcare databases, while other societal costs originate from Swedish studies published in the late 1990s and the 2000s.

The IHD costs are taken from a study that reported costs for angina pectoris patients. If no appropriate Swedish data on a cost item was found, the cost was assumed 0. The cost items are valued as reported, mostly as a point estimate, and usually the average cost across the patient group. If reported, the excess costs because of respective disease are used. The only stochastic costs are the CHF community care costs, which are based on the proportion of patients that consumed the care and the average costs for those patients, and the medical treatment costs, which are sampled as a uniform distribution from the 95 % confidence interval (CI) of costs, with different uniform distributions to prevent correlation. The sampling leads to a model mean cost that is slightly higher than the observed mean cost.

### 3.2.1 Medical treatment cost

The medical treatment costs are taken from all health care provided within the medical care system (in Swedish: landsting) to the population of Stockholm County (inhabitants 2004 1.8 million). The Stockholm County Council maintains a set of administrative databases (the VAL databases), partly created for the internal transactions between the providers and purchasers within the County Council. In the databases, all individuals in the County are assigned an individual identification number, which enables tracking the dates of migration and death and healthcare visits for all individuals, since the middle of the 1990s. In some databases, the individual’s age, gender and residential area are also recorded. Access to the database is restricted to employees of the Stockholm County Council.

Table 20. Standard costs for primary care. In current year SEK.

|  | **2002** | **2003** | **2004** | **2005** | **2006** |
| --- | --- | --- | --- | --- | --- |
| GPs | 1 030 | 1 120 | 1 150 | 1 100 | 1 120 |
| Nurses | 280 | 280 | 290 | 330 | 336 |
| Physiotherapists | 320 | 350 | 370 | 370 | 377 |

Source: Swedish Association of Local Authorities and Regions, 2002, 2003, 2004, 2005, 2006.

The inpatient and outpatient costs are taken from the diagnosis-based DRG-system data-base. The database registers every healthcare episode along with diagnoses (maximum of 10) based on ICD-10 (after year 1997) as well as data on the healthcare provider and patient. The database also records each episode’s DRG-points and the DRG-cost, complemented with reimbursements for extraordinary resource use, e.g. intensive care. The inpatient and outpatient costs are thus based on administratively set prices.

The primary care visits are also taken from the Stockholm County Council database, but as they are not included in the DRG-based system most of them are not priced. Some of these visits are instead valued by a Swedish standard cost, taken from the Swedish Association of Local Communities and Regions. Standard costs were available for general practitioners, nurses and physiotherapists, see table 20.

The costs are based on the medical treatment consumption of all individuals that consumed any inpatient or outpatient care with recorded metabolic disease ICD-diagnoses (see table 2), as any diagnosis up to eighth diagnosis, during the five-year period 2002 to 2006. All healthcare consumption (inpatient, outpatient and primary care) for these individuals during the five years were retrieved from the databases. To obtain the accurate healthcare consumption for respective disease, a number of exclusion criteria are used, based on population data and recorded ICD-diagnoses (up to eighth diagnosis) for the individuals. Note however that a large share of outpatient and almost all primary care visits lack a diagnosis. Only the consumption for individuals aged over 35 years is included.

The medical treatment costs are divided into initial costs, annual costs, and death costs. Initial costs are only relevant for the diseases with an acute phase which might induce high costs, i.e. AMI and stroke. Initial costs are the mean accumulated costs during six months after the first event of AMI or stroke during the time period 2001-

Table 21. Medical treatment cost. In SEK 2004.

|  | **Original data** | | | **Bootstrapped** | | | | |
| --- | --- | --- | --- | --- | --- | --- | --- | --- |
|  | **N** | **Mean** | **Median** | **95 % CI** | | | **Min** | **Max** |
| *Initial* |  |  |  |  |  |  |  |  |
| AMI | 16 061 | 114 087 | 86 910 | 106 285 | - | 122 911 | 102 054 | 131 258 |
| Stroke | 18 850 | 93 398 | 54 174 | 85 800 | - | 105 130 | 79 710 | 117 240 |
| *Annual* |  |  |  |  |  |  |  |  |
| AMI | 2 670 | 58 740 | 37 553 | 54 853 | - | 62 952 | 51 982 | 65 607 |
| IHD | 91 129 | 38 150 | 14 592 | 34 250 | - | 42 960 | 32 584 | 46 478 |
| CHF | 58 313 | 50 206 | 25 902 | 45 869 | - | 55 768 | 14 412 | 60 602 |
| Stroke | 6 600 | 45 600 | 14 252 | 41 084 | - | 50 888 | 39 383 | 54 792 |
| Diabetes | 10 496 | 32 744 | 8 852 | 28 800 | - | 37 037 | 26 286 | 40 968 |
| Macro complications | 7 667 | 98 291 | 72 386 | 91 931 | - | 105 476 | 88 516 | 110 183 |
| Micro complications | 3 556 | 28 849 | 4 296 | 23 666 | - | 35 140 | 19 894 | 38 786 |
| Both complications | 7 863 | 89 261 | 26 378 | 78 820 | - | 100 640 | 69 979 | 109 261 |
| *Death* |  |  |  |  |  |  |  |  |
| AMI | 387 | 194 868 | 157 985 | 185 013 | - | 206 782 | 177 389 | 215 260 |
| IHD | 3 144 | 203 070 | 153 043 | 190 528 | - | 217 396 | 178 436 | 225 577 |
| CHF | 6 764 | 174 682 | 134 631 | 164 004 | - | 187 082 | 160 668 | 195 062 |
| Stroke | 340 | 179 421 | 137 767 | 169 523 | - | 189 899 | 163 115 | 198 269 |
| Macro complications | 974 | 137 419 | 99 183 | 126 750 | - | 148 998 | 118 181 | 155 539 |
| Micro complications | 94 | 145 107 | 115 447 | 134 747 | - | 158 415 | 127 540 | 166 586 |
| Both complications | 842 | 143 925 | 117 539 | 109 956 | - | 167 116 | 85 061 | 177 686 |

2006, for the patients that survived these first six months. The death costs are the mean accumulated costs during six months before death, for patients with at least two visits with the respective diagnosis during the six-month period.

In the annual costs, the healthcare consumption during the year of death is excluded. If an individual is resident in Stockholm County for less than 6 month during a year, that year’s consumption is excluded. For the diseases that carry initial costs, i.e. AMI and stroke, the healthcare consumption during the first year is excluded. The costs of diabetes are divided into consumption because of only diabetes, for either micro or macro complications, or for both complications. At least two recorded diagnoses for diabetes are required during the year to classify the diabetes-related healthcare consumption as diabetes. Additionally, one visit with a recorded complication diagnosis is required to be included into the micro or macro complication groups, and at least two diagnoses from each complication group to be included into the both complications group. Only visits with a recorded diagnosis for respective disease is included in the annual health care costs. Note that the number of individuals, i.e. N, for the annual costs in table 21 concerns the number of person-years.

The medical treatment costs are mean costs over the ages (over age 35 years) and genders, see table 21. In the model, the medical treatment costs are sampled from the bootstrap results, as a uniform distribution. The bootstrap, with 1 000 replicates of 1 000 samples, were performed in the SAS statistical program (SAS Institute, 2007). The confidence interval (CI) was contructed from the 97,5% and 2,5% percentiles (Briggs et al, 1997), without adjustments to the interval limits. Medical treatment costs for sudden death are not included.

### 3.2.2 Other societal costs

*Pharmaceutical costs*

Pharmaceutical costs in Sweden ought to be divided between the County Councils and the patients, as patients pay a considerable share in co-payment. This is however not possible, given the data available. Table 22 therefore only presents the prescription pharmaceutical costs to the County Councils. The diabetes pharmaceuticals costs are the reported average costs over all diabetes patients, while the diabetes complications costs, used for any complication or both, were taken from

Table 22. Pharmaceutical costs. In SEK 2004.

|  | **annual cost** | **comment** | **source** |
| --- | --- | --- | --- |
| AMI | 3 700 | prescribed after event | Zethraeus et al, 1999 |
| IHD | 2 700 | angina pectoris-related | Andersson & Kartman, 1995 |
| CHF | 3 000 |  | Rydén-Bergsten & Andersson, 1999 |
| Stroke | 3 400 |  | Claesson et al, 2000 |
| Diabetes | 7 200 | all diabetics | Henriksson et al, 2000 |
| Diabetes complications | 10 300 | patients with both complications | Henriksson et al, 2000 |

Table 23. Costs for community care and technical aids. In SEK 2004.

|  | **annual cost** | **comment** | **source** |
| --- | --- | --- | --- |
| AMI | - |  |  |
| IHD | age <65 2 800  >65 7 400 | angina pectoris-related; social services and aids | Andersson & Kartman, 1995 |
| CHF | 4% of patients; 292 000 | nursing homes only | Rydén-Bergsten & Andersson, 1999 |
| Stroke | 8 500  2 200  35 700 | home adaptations and aids  transportation and aids  social services; difference year before and two years after disease onset | Gosman-Hedström et al, 2002  Claesson et al, 2000; von Koch et al, 2001  Ghatnekar et al, 2004 |
| Diabetes | 1 100 | excess costs; nursing homes only | Norlund et al, 2001 |

patients with both complications. The diabetes pharmaceuticals costs might therefore be somewhat overestimated.

*Costs for community care and technical aids*

These costs are paid by the municipalities in Sweden, and might include social services such as nursing homes and other seniors´ accommodation, rehabilitation, home assistance, transportation, technical aids and modifications at home. The costs are most comprehensive for stroke patients, see table 23, and lacking altogether for AMI.

Table 24. Costs for informal care and other costs for patients and relatives. In SEK 2004.

|  | **annual cost** | **comment** | **source** |
| --- | --- | --- | --- |
| AMI | - |  |  |
| IHD | age <65 4 600  >65 2 200  600 | angina pectoris-related; travel and time costs for contacts with health care services  angina pectoris-related; informal care for contacts with health care services | Andersson & Kartman, 1995 |
| CHF | - |  |  |
| Stroke | 12 000 | informal care, 5.6 hours/week at 42 SEK/hour | von Koch et al, 2001; Claesson et al, 2000 |
| Diabetes | 8 100 | excess costs; formal and informal home help | Norlund et al, 2001 |

Table 25. Productivity costs. In SEK 2004.

| **annual cost** | | **comment** | **source** |
| --- | --- | --- | --- |
| AMI | 115 000 | mean difference the year before and after disease onset | Zethraeus et al, 1999 |
| IHD | 107 000 | angina pectoris-related | Andersson & Kartman, 1995 |
| CHF | 27 000 | mean difference the year before and after disease onset | Zethraeus et al, 1999 |
| Stroke | 80 000 | mean difference the year before and after disease onset | Zethraeus et al, 1999 |
| Diabetes | 17 000 | excess cost | Norlund et al, 2001 |

*Costs for informal care and other costs for patients and relatives*

The costs of informal care include the value of the care given to patients by relatives and friends. The valuation of informal care (often supplied by old-age pensioneers outside the formal labour force) is disputed (Koopmanschap et al, 2008), and indeed valued in two different ways in the data sources used. The costs for IHD and stroke are valued according to the opportunity costs method, with a valuation of 35% of the average wage, while the costs for diabetes are valued with the proxy good method, using the average salary for nurses in municipalities. The diabetes informal care costs include both formal and informal care, with relatives supplying the majority. The costs are used in all diabetes health states, regardless of complication. Estimates of informal care costs could only be found for stroke, diabetes and partly for IHD, see table 24, but they probably constitute a sizeable part of total societal costs.

Other costs for patients and relatives are underestimated, as only some costs for transportation to health care could be included for IHD. Other possible costs could be patient fees for health care, pharmaceuticals and social services, as well as purchases of technical aids etc.

*Productivity costs*

The productivity costs include lost production because of morbidity, not mortality, before the age of 65 years, the customary Swedish age of retirement. The costs are valued by the human capital method, and thus only include losses in salaried work. The costs might be overestimated for AMI, CHF, and stroke, see table 25, if individuals after the acute phases of first disease occurrence return to previous levels of labour market participation during subsequent years.

## 3.3 The health effects

The health effects are measured as QALYs (quality-adjusted life-years) and YLS (life-years lost). QALYs are only presented discounted (3%) and YLS only undiscounted. All quality-of-life (QoL) weights are based on the EQ-5D instrument (Rabin & Charro, 2001) with population preference-based weights solicited via the TTO (time trade-off) method (Dolan, 2001).

The Swedish average population QoL weights for different age and gender-groups are taken from a study on the representative adult population in Stockholm County (Burström et al, 2001a). These QoL weights are used to represent the QoL of individuals without the model diseases, see table 26.

The QoL losses for CHD and stroke are taken from a US study on the marginal disutility of a large number of chronic diseases (Sullivan et al, 2005), while the QoL losses for diabetes and complications are taken from a Dutch study on type-2 diabetes patients (Redekop et al, 2002). The US study reports two disease weights; one obtained from individuals who had had a disease at any time in the past (called QPC), and another (CCC) from individuals that experienced the disease during participation in the study, i.e. recently. Following the author recommendations, we use the QPC disease valuation for the long-term QoL effects, called annual in table 27, and the CCC for the acute phases of AMI and stroke, i.e. during the first year after the first occurrence of disease, called initial. The CHF loss is taken from QPC Coronary heart disease while the IHD loss is assumed reflected by QPC Other heart disease. The diabetes study was part of CODE-2, thus classifying the diabetes complications in the same way as the rest of the present model. Note that

Table 26. Average Swedish population quality-of-life weights.

| **Age group** | **Men** | **Women** |
| --- | --- | --- |
| 20-29 | 0.91 | 0.88 |
| 30-39 | 0.90 | 0.86 |
| 40-49 | 0.86 | 0.85 |
| 50-59 | 0.84 | 0.82 |
| 60-69 | 0.83 | 0.78 |
| 70-79 | 0.81 | 0.78 |
| 80-88 | 0.74 | 0.74 |

Source: Burström et al, 2001a

Table 27. Disease-specific quality of weight losses.

| **Health state** | **QoL loss** | **Source** |
| --- | --- | --- |
| *Initial* |  |  |
| AMI | -0.03 | Sullivan et al, 2005 |
| Stroke | -0.05 | Sullivan et al, 2005 |
| *Annual* |  |  |
| AMI | -0.01 | Sullivan et al, 2005 |
| IHD | -0.01 | Sullivan et al, 2005 |
| CHF | -0.02 | Sullivan et al, 2005 |
| Stroke | -0.04 | Sullivan et al, 2005 |
| Diabetes | -0.00 | Redekop et al, 2002 |
| Macro complications | -0.08 | Redekop et al, 2002 |
| Micro complications | -0.07 | Redekop et al, 2002 |
| Both complications | -0.17 | Redekop et al, 2002 |

diabetes without complications is not expected to lead to any decrease in QoL, in accordance with the study results (Redekop et al, 2002). The Swedish and Dutch studies used the UK tariff to value the EQ-5D health states (Dolan, 1997), while the US study used the US tariff (Shaw et al, 2005).

All QoL valuations in the health states are based on the age group and gender-specific average QoL, from table 26, decreased with the respective marginal QoL effect of the disease, in table 27. The QoL of individuals in the health state At risk is assumed the same as the average population QoL. The initial decrements in QoL for AMI and stroke are assumed to reflect the acute phase of the first occurrence of an AMI and stroke, and modelled as transitional decreases. The annual decrements in QoL are used during time spent in the health states, and reflects QoL effects during subsequent years for AMI and stroke, and the chronic health effects of the other diseases.

3.4 Sensitivity analyses

All model parameters are varied in univariate sensitivity analyses. One set of analyses seek to incorporate the effects of obesity and physical activity on overall death risks and quality-of-life, to investigate if those patient and intervention characteristics affect the model estimates. In a multivariate analysis, the changes in model estimates introduced by only including previous Swedish data sources in the model is investigated. Some analyses on methodological choices are also performed, as well as a probabilistic analysis. The analyses are performed on men and women aged 50 years with all risk factor at low levels and at all high levels, respectively.

### 3.4.1 Univariate analyses

*A. Disease risks*

As all base case disease risks are taken from international studies, one analysis uses available Swedish disease risks. Risks for AMI and Stroke are taken from the NORA study (Dobson et al, 1998) as used previously (Löfroth et al, 2006). The risks for Diabetes are based on data from the SDPP (Stockholm Diabetes Prevention Program), a cohort study with 8 (women) and 10 (men) year follow-up (pers. com. A. Hilding, Karolinska Institutet, 2008-04-24). The risks for diabetes complications are not changed.

*B. Death risks*

The only sensitivity analysis on the death risks considers the effects of physical activity on relative risks of death, as the model might be used for interventions aiming to increase levels of physical activity. The average age- and gender-specific death risks, i.e. in the health state At risk, is assumed decreased by 33% if individuals are increasing their physical activity from the lowest level to the highest (Nocon et al, 2007). The disease death risks are not altered.

*C. Medical treatment costs*

The analysis on medical treatment costs replaces all Stockholm County Council costs with costs obtained from the Swedish studies from which the base case societal costs are taken, i.e. from Zethraeus et al (1999), Andersson & Kartman (1995), Rydén-Bergsten & Andersson (1999), and Henriksson et al (2000). The medical treatment costs for stroke are taken from Ghatnekar et al (2004), divided into first year costs and second and following years´ costs, where the latter are the undiscounted average costs from the reported discounted 2-4 year costs. As no measure of variability was reported in the studies, the point estimates are used.

*D. All costs*

As there are no alternative reports of Swedish disease-specific costs available, to our knowledge, the base case estimates are increased and decreased by 25%, to investigate the sensitivity of disease costs.

*E. QoL weights*

A number of analyses are performed using alternative QoL (quality of life) weights, to investigate several aspects of possible QoL effects. One analysis base all disease QoL weights on a Swedish study, which reported an average EQ-5D value of 0.60 for diabetes (all diabetics, regardless of complication status), 0.60 for IHD (used for all CHD) and 0.44 for stroke (Burström et al, 2001b). Another analysis incorporates QoL losses not considered in the base case; a decrease of 0.02 for diabetes without complications (Sullivan et al, 2005), and a decrease of 0.04 because of obesity (defined as BMI 30+) regardless of disease status (Sach et al, 2007).

Another aspect is the type of interventions that the model might be used for, in particular interventions to increase physical activity. The base case does not take account of any effects from an increased physical activity apart from effects on risk factor levels, but a number of studies (e.g. Laforge et al, 1996; Saavedra et al, 2007; Shibata et al, 2007) have shown that increased levels of physical activity affect QoL, as measured by the instrument SF-36. To our knowledge, however, there are no estimates on the effect of physical activity on composite QoL, why we assumed that an increased physical activity leads to an increase of 0.03 (which might be the minimally clinically important difference of the EQ-5D (Sullivan et al, 2005)), regardless of disease status.

Yet another analysis replaces the disease-specific deductions from average QoL with the average QoL loss of 0.05 for individuals that have the metabolic syndrome (including a BMI of 30+) and related diseases (Sullivan et al, 2007). The deduction is also applied in the At risk health state. This analysis seeks to consider that the model might be used for interventions that target persons that suffer from several risk factors for disease simultaneously, and thus might experience QoL losses not only from disease but also from the risk factors, such as obesity and high blood pressure.

### 3.4.2 Multivariate analyses

*F. Swedish data sources*

This analysis only includes previously published Swedish data, using the same data as in the analyses on disease risks (A), medical treatment costs (C) and parts of QoL weights (E).

### 3.4.3 Methodological aspects

*G. Discount rate*

Analyses using the discount rate 5% as well as no discounting at all, e.g. 0%, are performed, according to recommendations (LFN, 2003).

*H. Costs in added life years*

One sensitivity analysis investigates the effects of including costs in added years of life, i.e. the value of market production net of market consumption during saved life years, as recommended by LFN (2003). The age-specific data is taken from LFN (www.lfn.se).

*I. Termination age*

To investigate the effect of the termination age, the base case age of 85 years is changed to 120 years, which means that all individuals are dead at the end of the simulations. However, the disease risks are only applied until the age of 85 years, as the risks are considered too uncertain after that age. Thus, the individuals remain in the health state they were in at age 85 until death, and health effects and costs accumulate until death.

### 3.4.4 Probabilistic sensitivity analysis

A bootstrap analysis is performed using the Monte Carlo simulations of 10,000 repetitions for the groups men and women aged 50 years with all risk factors at high levels and at low levels, respectively. A sample of 1,000 repetitions from each simulation is drawn, with replacement, in the SAS version 9.2 (SAS Institute, Inc) and the mean of the costs and QALYs is calculated. This is replicated 1,000 times. The bootstrap is represented as a scatterplot in the cost-effectiveness plane. A “95% confidence interval” is calculated by the percentile method (Briggs, 2001), i.e. the tails of 2.5% of the distributions of the estimates of costs and QALYs, respectively, are deducted, leaving 95% of the observed distribution. Note that no differences between groups are calculated for the analysis.

4. Model output

To illustrate the model outputs in terms of societal costs, QALYs and YLS ( life-years lost) simulations have been performed on some assumed risk factor levels for the two genders at two ages. More detailed estimates and sensitivity analyses are reported for selected groups.

4.1 Model estimates from risk factor levels

The model estimates in terms of societal costs, QALYs and YLS (life-years lost before the age of 85 years, undiscounted) for men and women at starting ages 35 and 50 years for different risk factor levels differ considerably, see table 28. When all the risk factors are set at low levels, as defined in table 5, the model estimates costs of around 100,000 SEK and 1 YLS per individual for both genders and ages. This implies that the model simulates that even at low risk levels some individuals will contract CVD or diabetes before the age of 85 years, which is estimated to cost around 100,000 SEK on average. The average age of death for this group, as reflected in the loss of around 1 life-year (YLS) before the age of 85 years, is higher than the reported Swedish life expectancy of 79 years for men and 83 years for women (SCB),

Table 28. Model estimates from selected risk factor levels. Costs in SEK 2004.

|  | **age 35** | | |  | **age 50** | | |
| --- | --- | --- | --- | --- | --- | --- | --- |
| **risk factor** | **Costs** | **QALYs** | **YLS** |  | **Costs** | **QALYs** | **YLS** |
|  | **Men** | | | | | | |
| all low | 110 796 | 19.31 | 1.47 |  | 105 861 | 14.57 | 1.19 |
| BMI high | 130 444 | 19.20 | 1.68 |  | 119 592 | 14.53 | 1.32 |
| HDL high | 234 297 | 18.74 | 3.62 |  | 210 115 | 13.98 | 2.85 |
| SBP high | 193 754 | 19.06 | 2.50 |  | 183 687 | 14.32 | 1.91 |
| FPG+ HbA1c high | 485 302 | 17.64 | 8,32 |  | 374 919 | 13.12 | 5.08 |
| chol high | 172 464 | 19.04 | 2.53 |  | 155 952 | 14.22 | 2.09 |
| all high | 800 721 | 14.61 | 19.50 |  | 621 295 | 10.63 | 12.24 |
|  | **Women** | | | | | | |
| all low | 110 665 | 19.28 | 1.00 |  | 100 040 | 14.73 | 0.66 |
| BMI high | 150 747 | 19.17 | 1.62 |  | 125 529 | 14.66 | 1.01 |
| HDL high | 229 706 | 18.82 | 2.86 |  | 200 368 | 14.34 | 1.86 |
| SBP high | 192 885 | 19.06 | 1.88 |  | 167 359 | 14.50 | 1.30 |
| FPG+ HbA1c high | 506 049 | 18.13 | 6.13 |  | 381 757 | 13.74 | 3.54 |
| chol high | 141 304 | 19.18 | 1.59 |  | 125 005 | 14.58 | 1.15 |
| all high | 876 386 | 15.00 | 18.15 |  | 663 196 | 11.05 | 11.55 |

as expected. The QALYs differ according to starting age, around 20 and 15 QALYs in the two age groups, and similar for men and women.

When all the risk factors are set at high levels, i.e. well above the thresholds for increased risks, estimated societal costs increase to around 800-900,000 for those aged 35 and to 600-700,000 SEK for the 50-year-olds. The life-years lost increase to around 19 years for those aged 35 years and to around 12 years for the aged 50 years, while the number of QALYs decrease to around 15 and 11, with a somewhat lower number for the males. The other simulations, with one risk factor at a time set at a high risk level, reveal the effects from each risk factor on metabolic syndrome-related costs and health effects. A high BMI affects costs and health to a very small extent (probably an underestimate), while the FPG and the HbA1c that affect the risk for diabetes and related complications, have a large impact, in particular on women.

Table 29. Estimated disease costs per cost item, men aged 50 years all low and all high. In SEK 2004.

|  |  | **CHD** | **Stroke** | **Diabetes** | **Macro compl** | **Micro compl** | **Both compl** | **Total** |
| --- | --- | --- | --- | --- | --- | --- | --- | --- |
| Health care | |  |  |  |  |  |  |  |
|  | all low | 21 341 | 20 749 | 9 931 | 76 | 2 602 | 70 | 54 767 |
|  | all high | 90 585 | 15 454 | 131 940 | 114 138 | 699 | 1 303 | 354 119 |
| Pharmaceuticals | |  |  |  |  |  |  |  |
|  | all low | 964 | 1 246 | 2 187 | 26 | 146 | 4 | 4 574 |
|  | all high | 4 861 | 995 | 29 095 | 6 948 | 251 | 94 | 42 244 |
| Community care | |  |  |  |  |  |  |  |
|  | all low | 1 313 | 17 008 | 334 | 3 | 16 | 0 | 18 675 |
|  | all high | 5 357 | 13 581 | 4 445 | 742 | 27 | 10 | 24 162 |
| Patient time and travel | | |  |  |  |  |  |  |
|  | all low | 439 | 0 | 0 | 0 | 0 | 0 | 439 |
|  | all high | 2 643 | 0 | 0 | 0 | 0 | 0 | 2 643 |
| Informal care | |  |  |  |  |  |  |  |
|  | all low | 88 | 4 399 | 2 461 | 21 | 114 | 3 | 7 086 |
|  | all high | 458 | 3 512 | 32 732 | 5 464 | 197 | 74 | 42 437 |
| Productivity costs | |  |  |  |  |  |  |  |
|  | all low | 9 172 | 9 272 | 1 841 | 3 | 32 | 0 | 20 320 |
|  | all high | 85 648 | 11 640 | 51 928 | 6 241 | 206 | 25 | 155 689 |
| **Total** | all low | **33 318** | **52 674** | **16 754** | **129** | **2 909** | **77** | **105 861** |
|  | all high | **189 552** | **45 183** | **250 141** | **133 534** | **1 380** | **1 506** | **621 295** |

4.2 Detailed model output

To illustrate the composition of the societal costs, the costs per disease divided into cost items are reported for four risk factor groups; men and women aged 50 years with all risk factors at low levels and high levels, respectively, see table 29 and 30. The difference in costs between the all low and all high groups is considerable, around 500,000 SEK. The largest cost differences are found for diabetes and the macro complications from diabetes, mainly consisting of costs for health care and productivity costs. Due to competing risks, the costs for stroke are actually lower for the all high groups than for the all low.

Table 30. Estimated disease costs per cost item, women aged 50 years all low and all high. In SEK 2004.

|  |  | **CHD** | **Stroke** | **Diabetes** | **Macro compl** | **Micro compl** | **Both compl** | **Total** |
| --- | --- | --- | --- | --- | --- | --- | --- | --- |
| Health care | |  |  |  |  |  |  |  |
|  | all low | 7 646 | 17 488 | 22 313 | 5 294 | 160 | 54 | 52 955 |
|  | all high | 52 295 | 11 531 | 191 476 | 134 179 | 1 380 | 2 810 | 393 672 |
| Pharmaceuticals | |  |  |  |  |  |  |  |
|  | all low | 388 | 1 082 | 4 923 | 305 | 57 | 3 | 6 759 |
|  | all high | 2 919 | 762 | 42 256 | 8 422 | 491 | 193 | 55 042 |
| Community care | |  |  |  |  |  |  |  |
|  | all low | 517 | 14 769 | 752 | 33 | 6 | 0 | 16 077 |
|  | all high | 3 967 | 10 393 | 6 456 | 899 | 52 | 21 | 21 788 |
| Patient time and travel | | |  |  |  |  |  |  |
|  | all low | 188 | 0 | 0 | 0 | 0 | 0 | 188 |
|  | all high | 1 737 | 0 | 0 | 0 | 0 | 0 | 1 737 |
| Informal care | |  |  |  |  |  |  |  |
|  | all low | 36 | 3 819 | 5 538 | 240 | 45 | 3 | 9 681 |
|  | all high | 295 | 2 688 | 47 538 | 6 624 | 386 | 152 | 57 683 |
| Productivity costs | | |  |  |  |  |  |  |
|  | all low | 4 113 | 6 465 | 3 733 | 65 | 5 | 0 | 14 380 |
|  | all high | 49 160 | 8 352 | 69 441 | 5 989 | 281 | 52 | 133 274 |
| **Total** | all low | **12 888** | **43 623** | **37 258** | **5 937** | **273** | **61** | **100 040** |
|  | all high | **110 373** | **33 725** | **357 167** | **156 114** | **2 590** | **3 227** | **663 196** |

Table 31. Costs per Swedish payer, men and women aged 50 years all low and all high. In SEK 2004.

|  | **Women** | |  | **Men** | |
| --- | --- | --- | --- | --- | --- |
|  | **all low** | **all high** |  | **all low** | **all high** |
| County councils | 59 714 | 448 715 |  | 59 341 | 396 363 |
| Local authorities | 16 077 | 21 788 |  | 18 675 | 24 162 |
| Patients and relatives | 9 870 | 59 420 |  | 7 525 | 45 080 |
| Society | 14 380 | 133 274 |  | 20 320 | 155 689 |
| **Total** | **100 040** | **663 196** |  | **105 861** | **621 295** |

As might be expected, the largest difference in costs between the risk factor groups for different institutions in the Swedish society is found for the County councils, which are responsible for all medical care and pharmaceuticals, see table 31.

Underlying these differences in costs for the risk factor groups, are differences in disease outcome, see figures 2 and 3. The model estimates considerable differences in metabolic disease incidence between the all high and all low risk factor groups, in particular in diabetes and macro complications. Around 80% of women and 70% of men in the all high are estimated to contract diabetes, and the majority are estimated to contract macro complications. Among men in the all high, around 20% are expected to contract a coronary heart disease (CHD), but for both men and women the incidence of stroke is expected to be lower in the all high groups than in the all low, because of competing risks. Note that even at the low risk factor levels, some individuals (out of 10,000 simulated) are estimated to contract metabolic diseases; in particular diabetes and stroke among women, and CHD and stroke among men.

***
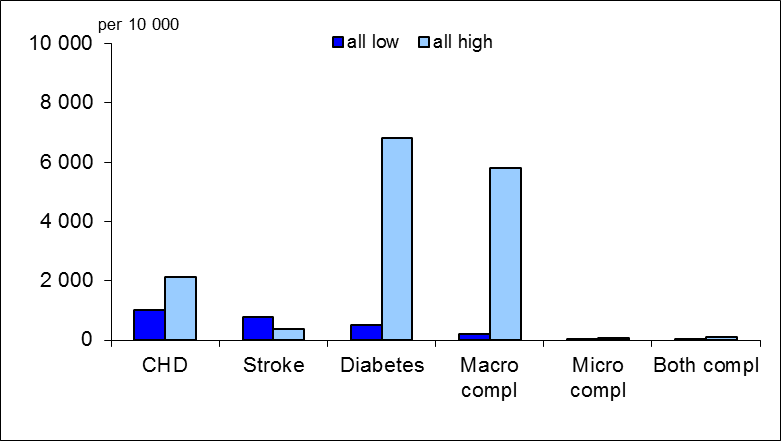

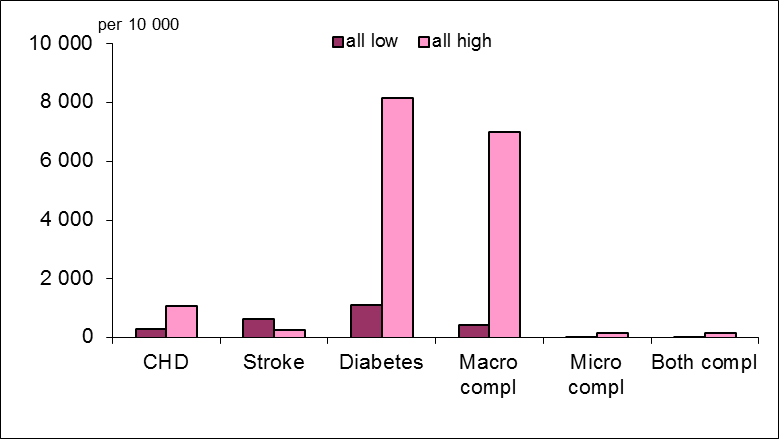
***

**Men Women**

Figure 2. Estimated incidence in metabolic diseases, men and women aged 50 years all low and all high. Per 10,000 modelled individuals.


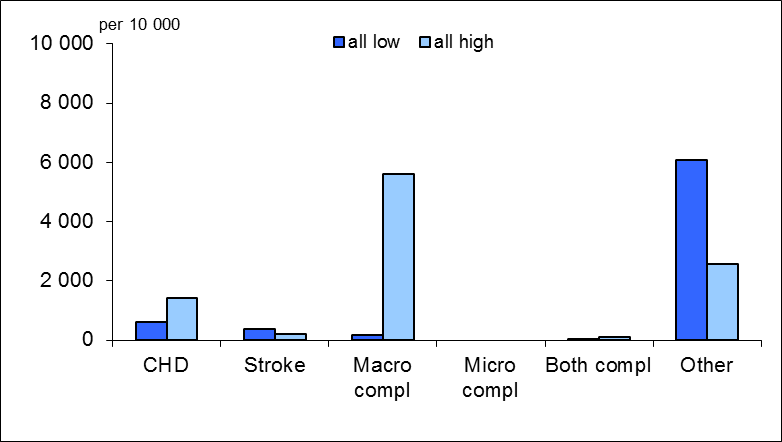

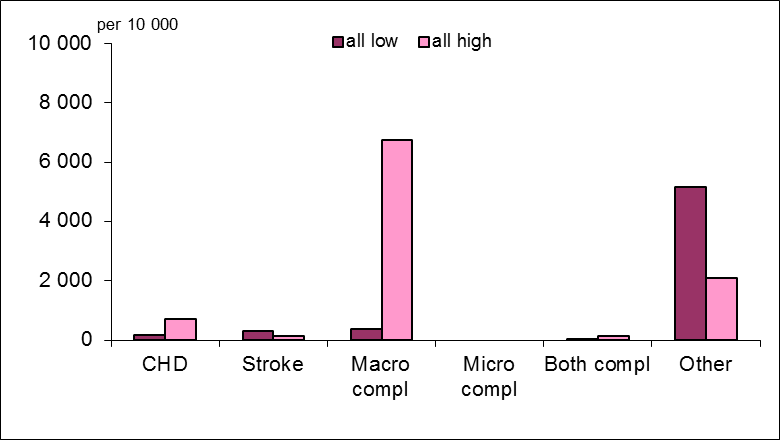


**Men Women**

Figure 3. Estimated mortality, men and women aged 50 years all low and all high. Per 10,000 modelled individuals.

The model estimates of mortality follows the same pattern as the morbidity, see figure 3. Almost all of the simulated individuals in the all high groups are expected to die before the age of 85 years, the majority of them in a metabolic syndrome disease, in particular macrovascular complications from diabetes. In contrast, around 40% of the women and 25% of the men in the all low risk factor groups are still alive at the end of the model simulations, i.e. at age 85 years. The predominant cause of death among them is Other causes, i.e. not related to metabolic disease.

4.4 Sensitivity analyses

Univariate, multivariate and methodological sensitivity analyses have been performed for four risk factor groups; men and women aged 50 years with all risk factors at low levels and high levels, respectively, see figure 4.

All alternative parameter choices affect the estimates for the all high groups the most, and the women somewhat more than the men. The cost estimates are considerably more sensitive to parameter choices than the QALY estimates. The methodology choices of discount rate (analysis G) and costs in added life years (H) influence the model estimates to the largest extent, where the inclusion of costs in added life years actually lead to cost savings for the all high groups. The multivariate analysis (F) that only includes Swedish data affects the groups differently; large decreases in costs and increases in QALYs for the all high groups, but increases in costs and decreases in QALYs for the all low men. This is a combined effect of in particular the alternative disease risks (A) and the medical treatment costs (C), even though some of the alternative QoL weights (E) are also used in the analysis.


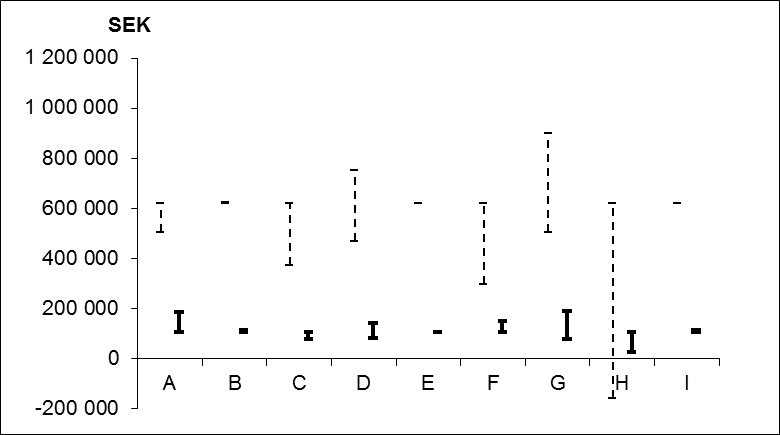

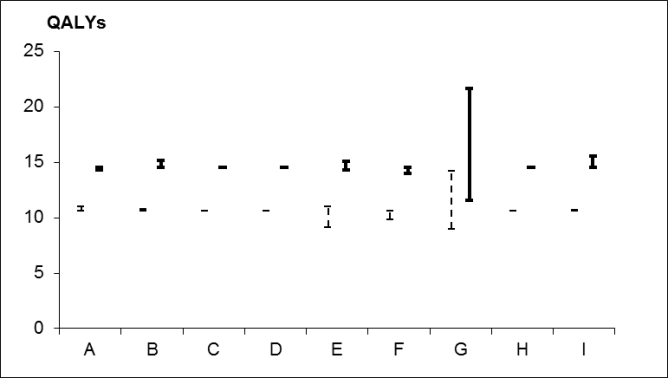


**Men**


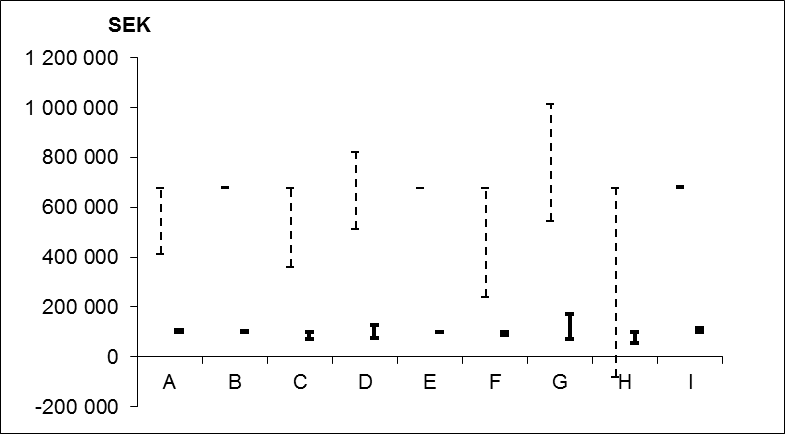

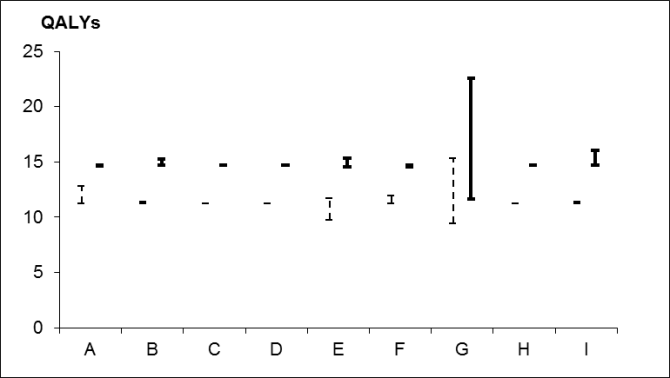


**Women**

A. Disease risks Swedish, B. Death risks physical active, C. Medical treatment costs, D. All costs +-25%, E. QoL weights, F. Swedish data sources, G. Discount rate 0% 5%, H. Costs in added life years, I. Termination age 120 years.

Figure 4. Sensitivity analyses on model parameters. Men and women aged 50 years at start with all low levels (bold) and high levels (dotted), in SEK 2004 and QALYs.

The alternative medical treatment costs (C) leads to considerable decreases in estimated disease-related costs, reflecting that the Stockholm County Council cost are considerably higher than previously reported cost estimates. Decreases in costs and increases in QALYs are expected when only Swedish disease risks (A) are incorporated in the model as the diseases IHD and CHF are excluded. Yet, for all low men the disease incidence increases, which leads to higher costs and lower QALYs than the base case. Among the sensitivity analyses on the QoL weights, the use of Swedish QoL weights leads to decreases in QALYs in particular for the all high group, while the inclusion of QoL effects from an increased physical activity increases the QALYs with about 0.5 QALYs in all groups. Including QoL effects from diabetes and obesity as well as from having the metabolic syndrome affects the QALY estimates to a lesser degree. Finally, changing the model termination age (I) and adjusting the death risks because of an increased physical activity (B) leads to very small changes in estimates.


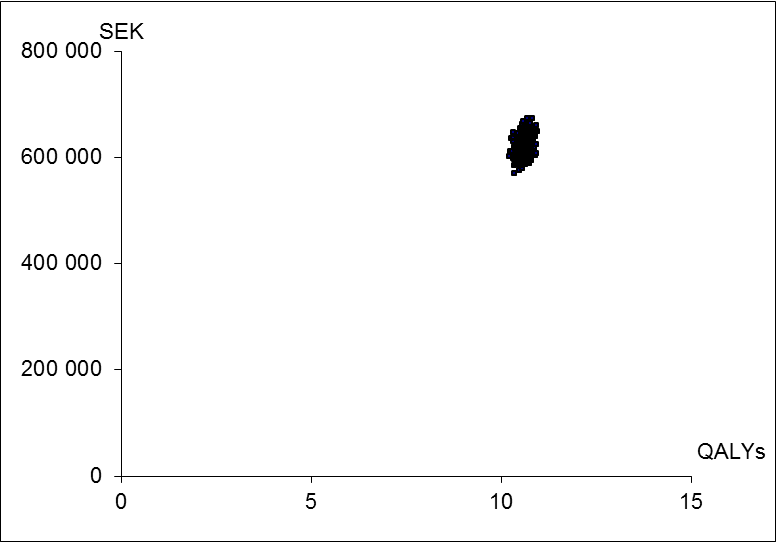


**all low Men all high**


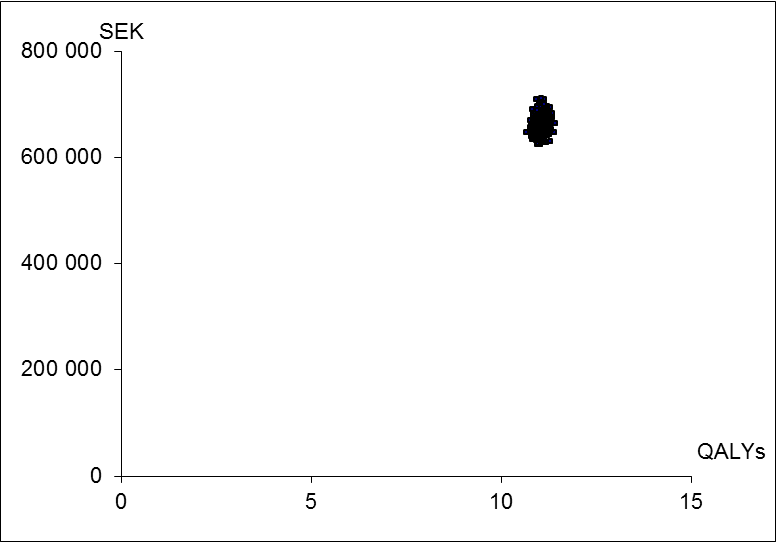


**all low Women all high**

Figure 5. Bootstraps from model simulations. Men and women aged 50 years at start with all risk factors at low levels and high levels. In SEK 2004 and QALYs.

The probabilistic sensitivity analysis, based on bootstraps of the 10,000 model repetitions, shows fairly consistent estimates, see figure 5. The cost estimates differ by about 100,000 SEK in the all high groups and 50,000 in the all low groups, while the differences between maximum and minimum QALY estimates are about 0.7–0.8 QALYs for all groups. The “95% confidence intervals” are narrower; 40-60,000 SEK and about 0.45 QALYs for all groups. There is no difference in uncertainty between the male and female groups.

5 Discussion

The revision of the metabolic syndrome model, and the technical report, sought only to update the diabetes part of the model. Apart from introducing the risk factor HbA1c, there are no other changes in the model. After reviewing the literature, the societal costs of diabetes or the diabetes-related diseases are not updated. The changes introduced entail only minor changes in the model results.

The validity of the model is discussed according to four aspects, as recommended by McCabe & Dixon (2000); the structure of the model, the inputs of the model, the results of the model and finally, the value of the model to the decision-maker.

5.1 The structure of the model

The aim of the model is to reflect the so-called metabolic syndrome, a clustering of lifestyle-related risk factors that affect the risks for cardiovascular disease and diabetes (Alberti et al, 2006), in order to enable cost-effectiveness analyses of interventions aimed to decrease these risk factors. The model thus simulates the metabolic syndrome-related societal costs and health effects for an individual with certain values of the risk factors, which can be used to value the consequences of interventions. A similar model has been constructed for the US (Caro et al, 2007), but this model seeks to reflect Swedish conditions.

The model includes the metabolic syndrome-related diseases diabetes and diabetes-related complications, CHD and stroke. The model thus contains 13 health states. The initial health state At risk reflects an individual without any metabolic syndrome-related disease, who is assumed to experience the average health-related quality-of-life of the gender and age group, to run the average gender and age group mortality risk and to incur no societal costs. The health state is termed At risk to indicate that the individuals modelled have elevated risk factor levels, which might impact on their quality-of-life and mortality risks, as assumed in one of the sensitivity analyses. The two different termination health states, i.e. Dead and Dead other, reflect that the modelled individuals run two different mortality risks; one disease-specific and one in other causes, i.e. Dead other. However, as the model diseases are very common causes of death, the average age- and gender-specific death risk is adjusted by the disease-specific risk (Zethraeus et al, 2004), to avoid overestimating the death risks and thus underestimating the societal costs.

The use of the Framingham risk function necessitates a division of CHD events into specific diseases, which has been performed differently in previous models (e.g. Weinstein et al, 1987; Johannesson et al, 1991; Lindgren et al, 2003; Johansson, 2004, Caro et al, 2007). In the present model, CHD is represented by three well-defined health states; acute myocardial infarction (AMI), congestive heart failure (CHF) and Sudden death, with a fourth health state, called Ischemic heart disease (IHD), containing remaining CHD conditions. The simplification was partly due to data requirement and partly to avoid excessive model complexity. Yet, the use of a health state that contains a variety of diseases and symptoms decreases the internal validity of the model, in particular as a large proportion of the patients contracting CHD will transition to the health state (around 50% of those below the age of 65 years and 25% of those older than 65 years).

There are several very elaborate diabetes models (The Mount Hood 4 Modeling Group, 2007 ), as well as models on specific diabetes-related complications (e.g Ragnarson Tennvall & Apelqvist, 2001; Palmer et al, 2007). As this model incorporates another disease group, the diabetes part of the model is simplified, to avoid excessive complexity. Diabetes is thus divided into four health states; Diabetes without complications, Macrovascular complications (mainly CVD and stroke but also peripheral vascular disease), Microvascular complications (renal failure, blind-ness and amputation) as well as Both complications simultaneously. The same division has previously been used in a large European study, the CODE-2 (Williams et al, 2002) and available data confirms that there are differences between the groups both in terms of costs (Henriksson et al, 2000; Williams et al, 2002) and QoL (Redekop et al, 2002). The risks to contract complications to diabetes are however calculated from data from another large study, the UKPDS (Clarke et al, 2004).

The model is Markov model appropriate for Monte Carlo simulations (also called micro simulation), constructed in a well-known data simulation programme (Treeage Inc). Markov models are deemed particularly suitable for diseases with a sustained risk over time and for chronic diseases (Sonnenberg & Beck, 1993; Kuntz & Weinstein, 2001; Drummond et al, 2005) and are extensively used for cost-effectiveness analyses (Drummond et al, 2005). Nevertheless, the Markov model inflexibility, i.e. the fixed time periods and in particular the mutually exclusive health states, affects the possibility of a true reflection of the disease progression for metabolic syndrome patients. In the model, individuals at risk might contract diabetes or CVD, and once the individual has fallen ill with diabetes, he/she might experience CVD as a complication to diabetes. However, individuals that contract CVD are not allowed to transition to the diabetes state, i.e. cannot contract diabetes. If individuals with a history of CVD and subsequent diabetes incurr higher societal costs and lower quality-of-life than CVD patients without later diabetes, then the model underestimates the true metabolic syndrome-related costs and health effects. It would have been possible to add some transition probabilities to the model to remedy this, e.g. to allow individuals within the CVD health states to transition to Macro complications. That health state would then be interpreted as concurrent diabetes and CVD. This might, however threathen the external validity of the model, as the costs, QoL and mortality risks for the health state are taken from studies on diabetic patients that contract different types of macro complications.

The model stages are one-year long, which is appropriate given the risk estimates used. No half-cycle correction was considered necessary, given the long time frame of the simulations and as the corrected results differ from the values obtained from the model trackers. The model termination age is 85 years, due to a lack of risk estimates for older ages. If the starting age of the simulated individuals is fairly high, the rather low maximum age of 85 years would tend to underestimate the differences in costs and health effects between risk factors levels. The sensitivity analysis that increased the termination age to 120 years (analysis I) had only marginal effects on costs but increased the estimated number of QALYs for in particular women in the all low risk group (with 1.3 QALYs).

5.2 The inputs of the model

Another aspect of the validity is the inputs of the model. The present model is fairly complex and contains a large number of parameters - however taken from comparatively few studies which affects the internal validity positively. The data has been chosen to reflect Swedish circumstances, with societal costs taken from avail-able Swedish studies. As the number of studies on the particular data items needed for the model is limited, no meta-analysis or other synthesis of data was performed.

The model seeks to comply with recommendations on the methodology of cost-effectiveness analysis (Gold et al, 1996; Drummond et al, 2005) and in particular Swedish recommendations (LFN, 2003). The model thus reflects the societal perspective, values morbidity productivity costs according to the human capital method but excludes mortality productivity costs, values health effects as QALYs, discounts future costs and effects with a 3% rate, and has been subjected to extensive sensitivity analyses, including a probabilistic analysis.

### 5.2.1 The risks

Even though the concept of a metabolic syndrome has existed for some decades there are still different clinical definitions used to classify patients (Alberti et al, 2006). A further complication for simulation models on the syndrome is that some clinical definitions are not included in validated risk functions, such as triglycerids and waist circumference. We therefore chose to dispense with the risk factor triglycerids and to replace the waist measure with BMI (body mass index), as BMI is included in the diabetes and the macro complications risk functions. In the CVD risk functions, no parameter that reflects overweight is included. The model thus does not fully reflect the most recent definition of the metabolic syndrome.

The disease risk estimates are taken from the most representative studies available, to our knowledge. The CHD and stroke risks are based on the Framingham risk function (Anderson et al, 1991), which has been used for a number of models predicting CHD and stroke (e.g. Johannesson et al, 1991; Risk Assessment Tool for Estimating 10-year Risk of Developing Hard CHD). The appropriateness of the Framingham risk function for contemporary European populations has been questioned and alternatives proposed (Wilhelmsen et al, 2004; Hippisley-Cox et al, 2007). One sensitivity analysis (A) thus replaced the risk functions with functions derived from Swedish populations, which decreased the metabolic disease incidence in the groups with high risk factors, but increased the incidence for the groups with low risk factor levels. The distribution between four groups of CHD disease: AMI, IHD, CHF and Sudden death was taken from Swedish statistics on diagnoses in inpatient care from the Hospital Discharge Register, 2005. The distribution depends on the age and gender of the patients.

The risk of developing diabetes was taken from the San Antonio Heart Study (Stern et al, 2002 ). This risk function includes all the factors of interest and reflects a very broad population in a well recognized study. Risks for diabetes-related macro and micro complications were derived from the large United Kingdoms Prospective Diabetes Study (UKPDS 68, Clarke et al, 2004). The UKPDS provides risks for macro complications: AMI, IHD, Stroke, CHF, as well as micro complications: Amputation, Blindness and Renal failure. The risks for macro and micro complications and the death risks from the macro complications, respectively, were computed as a risk for independent events, which probably leads to some underestimation.

Swedish national registers were used to estimate the majority of death risks and the distribution of CHD diseases:

*Swedish National Board of Health and Welfare Registers*

Registers from Swedish National Board of Health and Welfare are in general of a very high quality with almost 100% coverage. We use the following registers:

- Diagnoses in Inpatient Care
- Acute Myocardial Infarction (AMI) Statistics
- Cause of Death Statistics

Diagnoses in Inpatient Care is based on the Hospital Discharge Register. The register contains gender, age, home county and principal diagnosis according to ICD-10. The Swedish Hospital Discharge Register started in 1964, and covers all public inpatient care since 1987. The Hospital Discharge Register is updated annually with information from those who run health care since 1998. Nowadays, it is mandatory to report inpatient care as well as visits to the doctor in outpatient care – except for primary care. The coverage of the register is 100%.

Acute Myocardial Infarction Statistics include all persons aged 20 years and above who have had an acute myocardial infarction reported to the Hospital Discharge Register or the Cause of Death Register since 1997. All principal and secondary diagnoses with the ICD-9 code 410 and ICD-10 codes I21 and I22 are included. For some cases, the data has been supplemented with deaths with other ischaemic heart disease as the underlying cause of death. The coverage of the register is 100%.

The Swedish National Board of Health and Welfare Statistical Databases contain cause of death data since 1997. It reports the underlying cause of death coded according to ICD-10. For injuries, the external cause of injury is shown. The register includes all those who died during one calendar year and were registered in Sweden at the time of death, regardless of whether the death occurred inside or outside the country. The register does not include stillborns, persons who died on a temporary visit to Sweden or asylum seekers who have not yet obtained residence permits. Swedes who have emigrated and are no longer registered in Sweden are not included either.

*The National Quality Registers*

A system of National Quality Registers has been established in the Swedish health and medical services in the last decades. There are about 70 registries and four competence centres that receive central funding in Sweden**.** A national quality registry contains individualised data concerning patient problems, medical interventions, and outcomes after treatment; recorded for all healthcare production. It is annually monitored and approved for financial support by an Executive Committee.

We use the following Quality Registers:

- Riks-Stroke - The Swedish Stroke Register
- RiksSvikt - The Swedish Heart Failure Register

The Swedish Stroke Register (Riks-Stroke) covers approximately 80% of the patients with acute stroke. As of 1998, all hospitals in Sweden admitting patients with acute stroke report to the register (79 hospitals in 2008). The Swedish Heart Failure Register (RiksSvikt) covers approximately 50% of all the patients with a diagnosis of heart failure admitted to hospital or outpatient clinics. The register has 99 par-ticipating units (58 hospitals and 41 outpatient clinics) – almost 50% of all the clinics.

As the death risks are obtained from national disease-specific prevalence register, i.e. on a very large number of Swedish persons with a specific disease, the death risks obtained are very representative case fatality rates. As the registers only contain deaths within 12 months, only the death risk during the first year can be calculated. Death risks after the first year are instead assumed the same as the average age- and gender-specific death rates, which might underestimate the risk of death and hence overestimate the costs. On IHD there is no Swedish register, why the death risks were taken from the overview of 10-years results from a randomised trial by the Coronary Artery Bypass Graft Surgery Trialists Collaboration (Yusuf et al, 1994), probably leading to an overestimation of the risk as the health care technology has improved considerably since that study was performed. The risk was however used in a simulation model of the treatment of coronary heart disease (Cooper et al, 2002).

### 5.2.2 The costs

The societal costs are taken from Swedish studies, and if available, disease-related incremental costs. If accurately measured these costs represent costs that are avoided if an individual does not contract disease. However, the very frequent related and unrelated co-morbidity in the patient groups considered, make clear distinctions of costs for different diseases difficult, which might overestimate costs avoided.

The base case medical treatment costs were taken from the administrative system created for internal transactions in the Stockholm healthcare authority, which means that the prices cannot be assumed to reflect opportunity cost. However, the DRG-based system has existed for nearly ten years, with annual negotiations between purchasers and providers, which has lead to differential prices for the providers. Even though DRG-based costs are normally considered “shadow prices”, in Stockholm they are thus to some extent determined under market conditions, why they might approach opportunity cost. The quality of the data base is considered good. The possibility of acquiring a full account of disease-specific medical treatment consumption from a population of nearly 2 million people during five years was however deemed to offset the disadvantages; the costs reflect both current medical practice and related co-morbidity.

The related co-morbidity is reflected as the costs of a visit are included as long as the disease is any of the recorded diagnoses. This feature could imply an overestimate of costs, as one visit could be included in several disease-specific costs, but this is ruled out by the mutually exclusive health states. The method is actually partly offsetting this disadvantage, as related co-morbidity is included in the medical treatment costs, although unaccounted for in the Markov model structure.

The medical treatment costs were divided into initial costs (for AMI and stroke), annual costs and death costs, to avoid overestimating the average annual costs. The costs are however higher than most previously reported. The annual costs for AMI are about the same as the difference between first year and previous year costs, as reported by Zethraeus et al (1999), while the initial costs for AMI are considerably higher (initial cost is the appropriate comparison with the Zethraeus et al costs). The annual CHF medical treatment costs are also higher than the reported, but our initial stroke costs are considerably lower. The stroke initial costs are about the same as those reported by Claesson et al (2000) but lower than the first year costs in Ghatnekar et al (2004). The annual stroke costs are higher than the Ghatnekar et al (2004) estimates. The CHF annual costs are higher than previously reported (Rydén-Bergsten & Andersson, 1999; Agvall et al, 2005) while the IHD costs are somewhat lower than those reported for angina pectoris (Andersson & Kartman, 1995). The diabetes medical treatment costs in this study are also higher than previously re-ported (Henriksson et al, 2000; Norlund et al, 2001), in particular the macro complication costs (Henriksson et al, 2000). The differences in costs between the complication statuses are however similar to those reported by Henriksson et al (2000); lower costs for patients with micro complications than for those without complications. The diabetes costs, i.e. without complications, are in line with the average costs for diabetics in later studies (Ringborg et al, 2008).

Most previous studies are somewhat dated and might thus not accurately represent current disease costs, which might explain the higher Stockholm County Council

costs. Advances in medical technology might have increased medical treatment costs, as well as pharmaceutical costs, which in turn might have led to decreased disease severity and thus less care and productivity costs. The disease costs in the present model might thus be biased towards higher overall costs; current higher medical treatment costs might have led to lower other societal costs than reported in the older cost studies. The sensitivity analysis that replaced the medical treatment costs with previously reported costs (analysis C) indeed decreased the costs con-siderably, with about one third. Note however that much of this bias cancels out when two groups are compared in a cost-effectiveness analysis.

The other societal costs are taken from a number of Swedish studies published during the 1990 and 2000s. The costs for AMI are to a large extent underestimated, as there was only one Swedish study, to our knowledge, that has reported some cost items (Zethraeus et al, 1999). That study also covered the other cardiovascular diseases, but as the number of patients was small, only the AMI costs and the productivity costs which were reported as incremental costs, were used. The costs for IHD are taken from a study on patients with angina pectoris (Andersson & Kartman, 1995), i.e. only one of the diseases included in IHD, albeit the most frequent disease in the group. The costs for stroke and diabetes are the most fully represented, and include most societal costs, taken from two studies on diabetes (Henriksson et al, 2000; Norlund et al, 2001) but five different on stroke patients (Zethraeus et al, 1999; von Koch et al, 2001; Claesson et al, 2000; Gosman-Hedström et al, 2002; Ghatnekar et al, 2004 ). The internal validity of the stroke costs is thus questionable, but the patient group investigated is the same for at least two studies. Most diabetes costs are incremental costs, i.e. the costs for adult diabetic patients in excess of average patient costs, as reported (Norlund et al, 2001). These costs were however not reported by complication status, why the costs are assumed the same for all diabetes patients.

### 5.2.3 The health effects

The QoL weights are modelled as disease-related marginal decrements from the average population QoL. The weights are taken from three different studies; on a Swedish representative sample (Burström et al, 2001a), on Dutch diabetes type-2 patients (Redekop et al, 2002) , and on a US representative sample (Sullivan et al, 2005). All studies used the same health measure instrument, i.e. the EQ-5D, even though the European studies used the UK tariff and the US study used the US tariff to value the health states (Johnson et al, 2005).

The average QoL in different age groups and genders was thus taken from Swedish data while the reductions in QoL due to disease were taken from the international literature. There are Swedish reports on QoL for most of the model diseases (used in sensitivity analysis E) but these did not quite suit the model structure. We instead chose the option of few and suitable data sources, albeit from populations that might not be fully representative for Sweden. The CHD and stroke QoL data was chosen as the weights were collected with an identical method from a large representative population; between 20 000-37 000 individuals per year between years 2000-2002 (Sullivan et al, 2005). Further-more, the study enabled the use of different QoL weights during recent disease and long-standing disease. The diabetes QoL weights (Redekop et al, 2002) reported the QoL of diabetes-related complications, grouped in the same way as the remainder of this model (as the study was part of the CODE-2).

The unrepresentativeness of the QoL data is mitigated by the use of disease-specific decreases in QoL from the Swedish average population QoL. The marginal QoL decrements because of disease are thus assumed identical over the differing population groups, i.e. a constant disease-specific QoL decrease. The differing QALYs over the groups were instead achieved via the average age group and gender-specific QoL weights, from which the disease-specific QoL decrements were deducted. This assumes that even though the average health status differs between populations, the QoL effects from specific diseases do not. This might be untrue if the QoL effects from disease are determined also by social circumstances at the population level such as income distribution, welfare systems including healthcare systems, etc. However, the differences between populations in disease-specific QoL might be expected to be less than the differences in overall health-related QoL. Yet, the average population weights might also not be fully representative for the Swedish population, as they were obtained from a survey on the Stockholm County population. The weights have nevertheless been used in a large number of Swedish cost-effectiveness analyses.

In the model, the initial health state At risk carries no decrease in QoL. This might overestimate the QALY gains from reduced risk factor levels, as the populations for which the model is constructed, i.e. individuals that suffers from the metabolic syndrom, have risk factor levels that probably leads to some decreases in quality-of-life. They are often overweight and on medication. The population considered for the model is thus not the average Swedish population with the average Swedish age-specific QoL. Furthermore, the interventions for which the model estimates might be used often involve increased levels of physical activity. The effects from physical activity within the model is confined to changes in risk factor levels, which might underestimate the gains in QALYs from interventions. Studies show that an increased physical activity is correlated to increases in QoL (e.g. Laforge et al, 1996; Saavedra et al, 2007; Shibata et al, 2007) and to decreased mortality risks, that are not fully explained by the reduced risk factor levels. Finally, the health state diabetes carries no reduction in QoL in comparison with the average population QoL, according to the study used for the base case (Redekop et al, 2002). This is somewhat surprising, and is contradicted by other studies (e.g. Burström et al, 2001b; Sullivan et al, 2005). All these aspects are investigated in a set of univariate sensitivity analyses (analysis E) which showed that the use of available Swedish QoL weights and incorporating QoL effects from physical activity influenced the estimates the most.

In summary, the model estimates are influenced by the chosen model inputs, as reflected in the sensitivity analyses. However, the methodology choices, i.e. whether to include costs in added life-years (analysis H) and discount rate (analysis G) lead to the most marked changes, along with the multivariate analysis that only used available Swedish data sources (F). The methods employed do not reflect model uncertainty (Philips et al, 2006), whilst the actual purpose of models is to synthesize the most appropriate data available (Drummond et al, 2005), even if that data is taken from international sources instead of national. The sensitivity analyses indicate that parameter uncertainty is the least important source of uncertainty in estimates. Furthermore, as the model is run as a Monte Carlo simulation (micro simulation) of a large number of “individuals” with some parameters included as distributions (although mainly the medical treatment costs), some first and second order uncertainty (Briggs, 2000) is addressed already in the base case estimates. The bootstraps from the micro simulations, used for a probabilistic sensitivity analysis, also revealed rather narrow “95% confidence intervals”.

## 5.3 The results of the model

The third aspect of model validity is the results of the model, which often only can be discussed in relation to the results of other similar models (McCabe & Dixon, 2000). To our knowledge, there is only one previous model (Caro et al, 2007) that explicitly seeks to reflect the metabolic syndrome, i.e. a cluster of risk factors that lead to a concurrent risk of diabetes and CVD. There are several models that simulate diabetes progresssion (see The Mount Hood 4 Modeling Group (2007)), and some which simulates the risk of diabetes and related diseases from patients with impaired glucose intolerance (e.g. Hoerger et al, 2005), including one Swedish (Lindgren et al, 2007). As these last models include one of the criterias for the

Table 32. Comparison of model estimated outcomes with other models.

| **Outcome** | **Parameters** | **Present model** | **Other model** | **Source** |
| --- | --- | --- | --- | --- |
| 8-year risk of diabetes (%) | Age = 54  Sex = 0 (male)  Smok = 0  BMI =33  FG = 5,5  Chol =4,44  HDL = 0,99  SBP = 150  Diabetes = 0 | 25,6% | 25% | Framingham Offspring (Wilson et al, 2007) |
| 10-year risk of CHD as a complication to diabetes (%) | Age =60  Sex = 1 (female)  Smok = 1  BMI = 30  HbA1c = 5,5  Chol = 6  HDL =1  SBP = 150  Diabetes =1  YD=10 | 18,5% | 17,4% | UKPDS Risk Engine (Clark et al, 2004) |
| 10-year risk of stroke as a complication to diabetes (%) |  | 12,3% | 11,2% |  |
| 10-year risk of fatal CHD as a complication to diabetes (%) |  | 11,1% | 10,6% |  |
| 10-year risk of fatal stroke as a complication to diabetes (%) |  | 2,1% | 1,9% |  |
| 15-year cumulative incidence of CHF | Age =50  Sex = 0 (male)  Smok =0  BMI = 27,0  FG = 5,8  Chol = 7,1  HDL =1,2  SBP = 150  Diabetes =0 | 4,51% | 4,1% | Ingelsson et al, 2006 |

metabolic syndrome as well as similar diseases as the present model, these along with the Caro et al (2007) model would be the most appropriate for comparisons.

To validate the risk functions in the model, some representative variables were chosen to estimate outcomes from the present model and compared with the estimated outcomes from two well-known models and one Swedish epidemiological

study. The model estimated outcomes were very similar to the other estimates, see table 32. Furthermore, the diabetes risk function was tested using Swedish population data. The probability to develop diabetes for a person in a certain age group with average levels of BMI, FP-glucose, HDL-cholesterol and blood pressure corresponds very well with the incidence of diabetes in Sweden (Svedish National Population Survey, 2008).

The cost estimates are more difficult to validate. For a man aged 50 years with very high risk factor levels , i.e. the all high simulation, the average lifetime societal costs is estimated to 621,000 SEK. The costs per individual naturally differ with the disease contracted, from about 1.3 million SEK (discounted 3%) for a stroke patient to 340,000 SEK for a diabetic without complications, of which around 450,000 SEK and 185,000 SEK, respectively, are medical treatment costs.

The average costs per individual in the cost-effectiveness analysis reported by Lindgren et al (2007) is around 200,000 SEK, but the lower costs are expected as the Lindgren et al (2007) model includes fewer diseases. A US analysis of the DPP (Diabetes Prevention Program; Herman et al, 2005 using the model reported in Hoerger et al, 2005) reports average lifetime outcome medical costs of around 350,000 SEK (assuming a year 2005 currency exchange rate), i.e. also somewhat higher than the present model estimates. Yet another comparison might be with the Caro et al (2007) model simulations on US patients that fulfill the IDF criterias for metabolic syndrome, that resulted in 10 year healthcare-related costs of 280,000 SEK for stroke patients and 140,000 for diabetes patients (assuming a year 2005 exchange rate). These 10-year estimates seem more in line with the present model estimates. The patient groups in the studies mentioned above however have lower average risk factor levels than the assumed for this model´s simulations. In fact, the assumed risk factor levels in the all risk groups are extremely high, and would not appear in real patient groups, as pharmaceutical treatments would have lowered the levels considerably.

The plausibility of the cost estimates is thus better judged from estimates used for analyses of implemented interventions on real patient groups. The model has yet been used for two cost-effectiveness analyses in Sweden; one on a community-based diabetes prevention program in three communities in Stockholm (Johansson et al, 2009) and another on lifestyle modification in primary care patients in Kalmar County (Engman et al, 2009). In the community-based program, the average risk factor levels for the population aged around 55 years, i.e. after the 10-year project period, was estimated to lead to average lifetime societal costs of around 200,000 SEK per person. In the primary care patient group, with high risk factor levels, the changes in average societal costs after the one-year follow-up amounted to around 20,000 SEK. These estimates do not appear to be unrealistic.

## 5.4 The value of the model to the decision-maker

The fourth aspect of model validity is the usefulness of the model to make informed decisions. The aim of the model is to reflect Swedish conditions, to enable decisions based on cost-effectiveness analyses in a Swedish context. The purpose of models is not to reflect reality but to assemble information considered relevant for current day decisions, but it is difficult to judge whether a model is accurate enough for the decisions at hand. The model has however this far been used for two economic evaluations (Johansson et al, 2009; Engman et al, 2009).

From a public health perspective, a model that more closely reflects the outcomes of many programs directed towards overweight and physical activity would be more useful. There are examples of such models (Sörensen et al, 2005; Jacobs-van der Bruggen et al, 2007), but at this time we chose to construct a model that incorporate some biological risk factors that are related to these lifestyles. To the extent that implemented programs measure these biological risk factors among participants, the model can be used to estimate future health and societal cost consequences. Decision-makers should however be reminded that those estimates probably are underestimating the true health and cost effects from such lifestyle programs (Johansson, 2009).

6 References

Alberti KGMM, Zimmet P, Shaw J. Metabolic syndrome- a new world-wide definition. A consensus statement from the International Diabetes Federation. Diabet Med 2006; 23:469-480.

Agvall B, Borgquist L, Foldevi M, Dahlström U. Cost of heart failure in Swedish primary care. Scand J Primary Health Care 2005;23:227-232.

Anderson KM, Odell PM, Wilson PWF, Kannel WB. Cardiovascular disease risk profiles. Am Heart J 1991;121:293-298.

Andersson F, Kartman B. The cost of angina pectoris in Sweden. Pharmaeconomics 1995;8:233-244.

Briggs AH. Handling uncertainty in cost-effectiveness models. Pharmacoeconomics 2000;17:479-500.

Briggs AH. Handling uncertainty in economic evaluation and presenting the result. In: Drummond M, McGuire A, eds. Economic evaluation in health care- merging theory with practice. Oxford: Oxford University Press, 2001.

Briggs A, Schulpher M. An introduction to Markov modelling for economic evaluation. Pharmacoeconomics 1998;13:397-409.

Burström K, Johannesson M, Diderichsen F (2001a). Swedish population health-related quality of life results using the EQ-5D. [Qual Life Res](javascript:AL_get(this,%20'jour',%20'Qual%20Life%20Res.');) 2001;10:621-635.

Burström K, Johannesson M, Diderichsen F (2001b). Health related quality of life by disease and socio-economic group in the general population in Sweden. Health Policy 2001;55:51-69.

Caro JJ, O´Brien JA, Hollenbeak CS, Spackman E, Ben-Joseph R, Okamoto LJ, Paramore LC. Economic burden and risk of cardiovascular disease and diabetes in patients with different cardiometabolic risk profiles. Value in Health 2007;10:S12-S20.

Cooper K, Davies R, Roderick P, Chase D, Raftery J. The development of a simulation model of the treatment of coronary heart disease. Health Care Management Science 2002;5:259-267.

Clarke PM, Gray AM, Briggs A, Farmer AJ, Fenn P, Stevens RJ, Matthews DR, Stratton IM, Holman RR. A model to estimate the lifetime health outcomes of patients with Type 2 diabetes: the United Kingdom Prospective Diabetes (UKPDS) Outcomes Model (UKPDS no. 68). Diabetologica 2004;47:1747-1759.

Claesson L, Gosman-Hedström G, Johannesson M, Fagerberg B, Blomstrand C. Resource utilization and costs of Stroke Unit care integrated in a care continuum: A 1-year controlled, prospective, randomized study in elderly patients. Stroke 2000;31:2569-2577.

Dobson AJ, Evans A, Ferrario M, Kuulasmaa KA, Moltchanov VA, Sans S, Tunstall-Pedoe H, Tuomilehto JO, Wedel H, Yarnell J, for the WHO MONICA Project. Changes in estimated coronary risk in the 1980s: data from 38 populations in the WHO MONICA Project. Ann Med 1998;30:199-205.

Dolan P. Modeling valuations for EuroQol health states. [Med Care](javascript:AL_get(this,%20'jour',%20'Med%20Care.');) 1997;35:1095-1108.

Dolan P. Output measures and valuation in health. In: Drummond M, McGuire A, eds. Economic evaluation in health care- merging theory with practice. Oxford: Oxford University Press, 2001.

Drummond M, McGuire A, eds. Economic evaluation in health - merging theory with practice. Oxford: Oxford University Press, 2001.

Drummond MF, Sculpher MJ, Torrance GW, O’Brien BJ, Stoddart GL. Methods for the economic evaluation of health care programmes. (3nd edition). Oxford: Oxford University Press, 2005.

Engman K-O, Feldman I, Hagberg L, Hellström L, Henriksson G, Johansson P. Hälsoekonomisk utvärdering av Metabola projektet i Kalmar län [Health economic evaluation of the Metabolic project in Kalmar County]. Oskarshamn: Landstinget i Kalmar Län, 2009. Available at www.ltkalmar.se and www.folkhalsoguiden.se

Ford ES. Risks for all-cause mortality, cardiovascular disease, and diabetes associated with the metabolic syndrome. Diabetes Care 2005; 28:1769-1778.

Ghatnekar O, Persson U, Glader E-L, Terént A. Cost of stroke in Sweden: An incidence estimate. Int J Technol Assess Health Care 2004;20:375-380.

Gold MR, Siegel JE, Russell LB, Weinstein MC, editors. Cost-effectiveness in health and medicine. New York: Oxford University Press, 1996.

Gosman-Hedström G, Claesson L, Blomstrand C, Fagerberg B, Lundgren-Lindquist B. Use and costs of assistive technology the first year after stroke. Int J Technol Assessmen Health Care 2002;18:520-527.

Henriksson F, Agardh C-D, Berne C, Bolinder J, Lönnqvist F, Stenström P, Östensson C-G, Jönsson B. Direct medical costs for patients with type 2 diabetes in Sweden. J Intern Med 2000;248:387-396.

Herman WH, Hoerger TJ, Brandle M, Hicks K, Sorenson S, Zhang P, Hamman RF, Ackermann RT, Engelgau MM, Ratner RE, for the Diabetes Prevention Program Research Group. The cost-effectiveness of lifestyle modification or metformin in preventing type 2 diabetes in adults with impaired glucose tolerance. Ann Intern Med 2005;142:323-332.

Hippisley-Cox J, Coupland C, Vinogradova Y, Robson J, May M, Brindle P. Derivation and validation of QRISK, a new cardiovascular disease risk score for the United Kingdom: prospective open cohort study. BMJ 2007;335:136.

Hoerger TJ, Hicks KA, Bethke AD. A Markov model of disease progression and cost-effectiveness for type 2 diabetes. Technical report accompanying Herman WH et al, 2005.

Ingelsson E, Ärnlöv J, Lind L, Sundström J. Metabolic syndrome and risk for heart failure in middle-aged men. Heart 2006;92;1409-1413.

Jacobs-van der Bruggen M, Bos G, Bemelmans WJ, Hoogenveen RT, Vijgen SM, Baan CA. Lifestyle interventions are cost-effective in people with different levels of diabetes risk. Diabetes Care 2007;30:128-134.

Johannesson, M, Hedbrant J, Jönsson B. A computer simulation model for cost-effectiveness analysis of cardiovascualar disease prevention. Linköping: Centrum för utvärdering av medicinsk teknologi (CMT), 1991. 1991:3.

Johansson P. A model for economic evaluations of smoking cessation interventions –tecnical report. Stockholm: Stockholm Centre for Public Health, 2004. Available at [www.folkhalsoguiden.se](http://www.folkhalsoguiden.se).

Johansson P. Economic evaluation of public health programmes – constraints and opportunities [thesis]. Stockholm: Karolinska Institutet, 2009.

Johansson P, Östenson C-G, Hilding A, Andersson C, Rehnberg C, Tillgren P. A cost-effectiveness analysis of a community-based diabetes prevention program in Sweden. Int J Technol Assess Health Care 2009;25:350-358.

Johnson JA, Luo N, Shaw JW, Kind P, Coons SJ. Valuations of EQ-5D health states. Are the United States and United Kingdom different? Med Care 2005;43:221-228.

Koopmanschap MA, van Exel NJA, van den Berg B, Brouwer WBF. An overview of methods and applications to value informal care in economic evaluations of healthcare. Pharmacoeconomics 2008;26:269-280.

Kuntz K, Weinstein M. Modelling in economic evaluation. In Drummond M, McGuire A, eds. Economic evaluation in health care -Merging theory with practice. Oxford: Oxford University Press, 2001.

Laforge RG, Rossi JS, Prochaska JO, Velicer WF, Levesque DA, McHorney CA. Stage of regular exercise and health-related quality of life. Prev Med 1999;28:349-360.

LFN [Swedish Pharmaceutical Benefits Board]. General guidelines for economic evaluations from the Pharmaceutical Benefits Board (LFNAR 2003:2). [www.lfn.se](http://www.lfn.se)

Lindgren P, Lindström J, Tuomilehto J, Uusitupa M, Peltonen M, Jönsson B, de Faire U, Hellénius M-L, The DPS Study group. Int J Technol Assessment in Health Care 2007;23:2.

Lindgren P, Fahlstadius P, Hellenius M-L, Jönsson B, de Faire U. Cost-effectiveness of primary prevention of coronary heart disease through risk factor intervention in 60-year-old men from the county of Stockholm – a stochastic model of exercise and dietary advice. Prev Med 2003;36:403-409.

Löfroth E, Lindholm L, Wilhelmsen L, Rosén M. Optimising health care within given budgets: Primary prevention of cardiovascular disease in different regions of Sweden. Health Policy 2006;75:214-229.

Malmberg K, Norhammar A, Wedel H, Rydén L. Glycometabolic state at admission: Important risk marker of mortality in conventionally treated patients with Diabetes Mellitus and Acute Myocardial Infarction. Long-term results from the Diabetes and Insulin-Glucose Infusion in Acute Myocardial Infarction (DIGAMI) Study. Circulation. 1999;99:2626-2632.

McCabe C, Dixon S. Testing the validity of cost-effectiveness models. Pharmacoeconomics 2000;17:501-513.

Nocon M, Hiemann T, Müller-Riemenschneider F, Thalau F, Roll S, Willich SN. Association of physical activity with all-cause and cardiovascular mortality: a systematic review and meta-analysis. Eur J Cardiovasc Prev Rehabil 2007:15;239-246.

Norlund A, Apelqvist J, Bitzén P-O, Nyberg P, Scherstén B. Cost of illness of adult diabetes mellitus underestimated if comorbidity is not considered. J Internal Med 2001;250:57-65.

Palmer AJ, Valentine WJ, Ray JA. Irbesartan treatment of patients with type 2 diabetes, hypertension and renal disease: a UK health economics analysis. Int J Clin Pract 2007;61:1626-1633.

Philips Z, Bojke L, Sculpher M, Claxton K, Golder S. Good practice guidelines for decision-analytic modelling in health technology assessment. Pharmacoeconomics 2006;24:355-371.

Rabin R, de Charro F. EQ-5D: a measure of health status from the EuroQol Group. Ann Med 2001;33:337-343.

Ragnarson Tennvall G, Apelqvist J. Prevention of diabetes-related foot ulcers and amputations: a cost-utility analysis based on Markov model simulations. Diabetologia 2001;44:2077-2087.

Redekop WK, Koopmanschap MA, Stolk RP, Rutten GEHM, Wolffenbuttel BHR, Niessen LW. Health-related quality of life and treatment satisfaction in Dutch patients with type 2 diabetes. Diabetes Care 2002;25:458-63.

RiksSvikt - The Swedish Heart Failure Registry. [www.ucr.uu.se/rikssvikt](http://www.ucr.uu.se/rikssvikt)

Riks-Stroke - **The Swedish Stroke** Registry.www.riks-stroke.org

Ringborg A, Martinell M, Stålhammar J, Yin DD, Lindgren P. Resource use and costs of type 2 diabetes in Sweden – estimates from population-based register data. Int J Clin Pract 2008;62:708-716.

Risk Assessment Tool for Estimating 10-year Risk of Developing Hard CHD. http://hp2010.nhlbihin.net/atpiii/calculator.asp

Rydén-Bergsten T, Andersson F. The health care costs of heart failure in Sweden. J Intern Med 1999;246:275-284.

Saavedra JM, de la Cruz, E, Escalante Y, Rodríguez FA. Influence of a medium-impact aquaerobic program on health-related quality of life and fitness level in healthy adult females. J Sports Med Phys Fitness 2007;4:468-74.

Sach TH, Barton GR, Doherty M, Muir KR, Jenkinson C, Avery AJ. The relationship between body mass index and health-related quality of life: comparing the EQ-5D, EuroQol VAS and SF-6D. Int J Obesity 2007;31:189-196.

SCB (Statistics Sweden). Statistics Database [www.scb.se](http://www.scb.se)

Shaw JW, Johnson JA, Coons SJ. US valuation of the EQ-5D health states. Development and testing of the D1 valuation model. Med Care 2005; 43:203-220.

Shibata A, Oka K, Nakamura Y, Muraoka I. Recommended level of physical activity and health-related quality of life among Japanese adults. HQLO (Health and Quality of Life Outcomes) 2007,5:64.

Sonnenberg FA, Beck JR. Markov models in medical decision making: A practical guide. Med Decis Making 1993;13:322-338.

Stern MP, Williams K, Haffner SM. Identification of people at high risk for type 2 diabetes mellitus: do we need the oral glucose tolerance test? Ann Intern Med 2002; 136:575-581.

Sullivan PW, Lawrence WF, Ghushchyan V. A national catalog of preference-based scores for chronic conditions in the United States. Med Care 2005;43:736-749.

Swedish Association of Local Authorities and Regions [Sveriges Kommuner och Landsting]. Activities and economy in county councils and regions [Verksamhet och ekonomi i landsting och regioner]. Stockholm: 2002, 2003, 2004, 2005, 2006.

Sörensen J, Horsted C, Andersen LB. Modellering af potentielle sundheds-ökonomiske konsekvenser ved öget fysisk aktivitet i den voksne befolkning [Modelling the potential healthcare economic consequences of increased physical activity in the adult population]. Odense: CAST, Syddansk universitet, 2005.

The Mount Hood 4 Modeling Group. Computer Modeling of Diabetes and Its Complications –A report on the Fourth Mount Hood Challenge Meeting. Diabetes Care 2007;30:1638-1646.

The Riksbank (Sveriges Riksbank). Exchange rates. [www.riksbank.com/templates/Page.aspx?id=17182](http://www.riksbank.com/templates/Page.aspx?id=17182)

The Swedish National Board of Health and Welfare. Cause of death in Sweden in 2007. Stockholm: The Board, 2009.

The Swedish National Board of Health and Welfare Statistical Databases. http://www.socialstyrelsen.se/en/Statistics/statsbysubject/

National Board of Health and Welfare. National Guidelines for Treatment and

Care of Diabetes Mellitus. Stockholm: National Board of Health and Welfare, 2010.

The Swedish National Population Survey, 2008. http://www.fhi.se/sv/Statistik-uppfoljning/Nationella-folkhalsoenkaten/

von Koch L, de Pedro-Cuesta J, Kostulas V, Almazán J, Widén Holmqvist L. Randomized controlled trial of rehabiliation at home after stroke: One-year follow-up of patient outcome, resource use and cost. Cerebrovasc Dis 2001;12:131-138.

Weinstein MC, Coxson PG, Williams LW, Pass TM, Stason WB, Goldman, L. Forecasting coronary heart disease incidence, mortality and cost: The Coronary Heart Disease Policy Model. Am J Public Health 1987;77:1417-26.

World Health Organisation. Definition and diagnosis of diabetes mellitus and intermediate hyperglycemia - Report of a WHO/IDF Consultation, 2006.

Wilhelmsen L, Wedel H, Conroy R, Fitzgerald T. Det svenska SCORE-diagrammet för kardiovaskulär risk [The Swedish SCORE diagram for cardiovascular risk]. Läkartidningen 2004;101:1798-1801.

Williams R, Van Gaal L, Lucioni C. Assessing the impact of complications on the costs of type II diabetes. Diabetologia 2002;45:S13-S17.

Wilson P, Meigs J, Sullivan L, Fox C, Nathan D, D’Agustino R. Prediction of incident diabetes mellitus in middle-aged adults. The Framingham Offspring Study. Arch Intern Med 2007;167:1068-1074.

Yusuf F, Zucker D, Peduzzi P, Fisher L, Takaro T, Kennedy J. Effect of coronary artery bypass graft surgery on survival: Overview of 10-year results from randomised trial by the Coronary Artery Bypass Graft Surgery Trialists Collaboration. Lancet 1999;344:563-570.

Zethraeus N, Molin T, Henriksson P, Jönsson B. Costs of coronary heart disease and stroke: the case of Sweden. J Intern Med 1999;246:151-159.

Zethraeus N, Borgström F, Jönsson B, Kanis J. A reassessment of the cost-effectiveness of hormone replacement therapy in Sweden – results based on the Women´s Health Initiative randomised trial. Stockholm: Stockholm School of Economics, SSE/EFI Working Paper Series in Economics and Finance, 2004. No 571.
